# Berberine Prevents Disease Progression of Nonalcoholic Steatohepatitis through Modulating Multiple Pathways

**DOI:** 10.3390/cells10020210

**Published:** 2021-01-21

**Authors:** Yanyan Wang, Yun-Ling Tai, Derrick Zhao, Yuan Zhang, Junkai Yan, Genta Kakiyama, Xuan Wang, Emily C. Gurley, Jinze Liu, Jinpeng Liu, Jimin Liu, Guanhua Lai, Phillip B. Hylemon, William M. Pandak, Weidong Chen, Huiping Zhou

**Affiliations:** 1 Department of Microbiology and Immunology, Medical College of Virginia and McGuire Veterans Affairs Medical Center, Virginia Commonwealth University, 1220 East Broad Street, MMRB-5044, Richmond, VA 23298, USA; aucmwyy@hotmail.com (Y.W.); Yunling.Tai@vcuhealth.org (Y.-L.T.); Derrick.Zhao@vcuhealth.org (D.Z.); 11718229@zju.edu.cn (Y.Z.); yanjunkai@xinhuamed.com.cn (J.Y.); xuan.wang@vcuhealth.org (X.W.); emily.gurley@vcuhealth.org (E.C.G.); phillip.hylemon@vcuhealth.org (P.B.H.); 2 School of Pharmaceutical Science, Anhui University of Chinese Medicine, Qianjiang, Hefei 230012, China; wdchen@ahtcm.edu.cn; 3 Department of Internal Medicine, Medical College of Virginia and McGuire Veterans Affairs Medical Center, Virginia Commonwealth University, Richmond, VA 23298, USA; genta.kakiyama@vcuhealth.org (G.K.); william.pandak@va.gov (W.M.P.); 4 Department of Biostatistics, Virginia Commonwealth University, Richmond, VA 23298, USA; Jinze.Liu@vcuhealth.org; 5 Department of Computer Science, University of Kentucky, Lexington, KY 40506, USA; lji226@uky.edu; 6 Department of Pathology and Molecular Medicine, McMaster University, Hamilton, ON L6M0L8, Canada; liuj107@mcmaster.ca; 7 Department of Pathology, Medical College of Virginia, 23298 Virginia Commonwealth University, Richmond, VA 23298, USA; guanhua.lai@vcuhealth.org

**Keywords:** NAFLD, bile acids, inflammation, fibrosis

## Abstract

**Background and Aims**: The disease progression of nonalcoholic fatty liver disease (NAFLD) from simple steatosis (NAFL) to nonalcoholic steatohepatitis (NASH) is driven by multiple factors. Berberine (BBR) is an ancient Chinese medicine and has various beneficial effects on metabolic diseases, including NAFLD/NASH. However, the underlying mechanisms remain incompletely understood due to the limitation of the NASH animal models used. **Methods**: A high-fat and high-fructose diet-induced mouse model of NAFLD, the best available preclinical NASH mouse model, was used. RNAseq, histological, and metabolic pathway analyses were used to identify the potential signaling pathways modulated by BBR. LC–MS was used to measure bile acid levels in the serum and liver. The real-time RT-PCR and Western blot analysis were used to validate the RNAseq data. **Results**: BBR not only significantly reduced hepatic lipid accumulation by modulating fatty acid synthesis and metabolism but also restored the bile acid homeostasis by targeting multiple pathways. In addition, BBR markedly inhibited inflammation by reducing immune cell infiltration and inhibition of neutrophil activation and inflammatory gene expression. Furthermore, BBR was able to inhibit hepatic fibrosis by modulating the expression of multiple genes involved in hepatic stellate cell activation and cholangiocyte proliferation. Consistent with our previous findings, BBR’s beneficial effects are linked with the downregulation of microRNA34a and long noncoding RNA H19, which are two important players in promoting NASH progression and liver fibrosis. **Conclusion**: BBR is a promising therapeutic agent for NASH by targeting multiple pathways. These results provide a strong foundation for a future clinical investigation.

## 1. Introduction

Nonalcoholic fatty liver disease (NAFLD) is the most common chronic liver disease worldwide. The prevalence of NAFLD has been continuously increasing during the last decade globally in the obesity epidemic. The disease progression from simple steatosis (NAFL) to nonalcoholic steatohepatitis (NASH), which can progress to fibrosis and cirrhosis, has been closely linked to inflammation, obesity, insulin resistance, and metabolic syndrome [1]. As NAFLD rapidly increased in frequency during the last decade, NASH-related liver failure is predicted to be the primary cause for liver transplantation in the coming years [2]. During the last several decades, extensive efforts have been made to identify the potential molecular/cellular mechanisms underlying NAFLD/NASH disease progression and to develop a therapeutic strategy. However, the pathogenesis of NASH progression remains incompletely understood and no effective therapy is currently available.

It is well accepted now that NAFLD is not a single-target disease and progression of NAFL/NASH is driven by multiple factors, such as dysregulation of hepatic lipid metabolism, activation of inflammation, endoplasmic reticulum (ER) stress, oxidative stress, and hepatic stellate cells (HSCs), as well as dysbiosis of the gut microbiota [1,2,3]. Therefore, an effective therapeutic agent must have the capacity to modulate multiple pathways involved in the NAFL/NASH progression.

Berberine (BBR) is an isoquinoline alkaloid and was originally isolated from the rhizome of the perennial herb *Coptis chinensis*. It has been widely used in traditional Chinese medicines for diarrhea and various infectious gastrointestinal disorders for more than 3000 years [4]. In the past decades, numerous studies reported that BBR has multiple beneficial effects on preventing cardiovascular and metabolic diseases, including NAFLD and diabetes [5,6,7,8]. Our previous studies also showed that BBR could inhibit human immunodeficiency virus (HIV) protease inhibitor-induced ER stress and tumor necrosis factor alpha (Tnfα) and interleukin (IL)-6 expression through regulating the RNA-binding protein (RBP) Hu antigen R (HuR) in macrophages [9]. We also reported that BBR exerts its lipid-lowering effect via modulating gut microbiome and bile acid metabolism in a high-fat diet (HFD)-induced hamster hyperlipidemia model [10]. Up to now, the published studies with BBR on NAFL/NASH used various HFD-fed mouse models or HFD-fed rat, hamster, or zebrafish models. These preclinical models fail to recapitulate human NAFL/NASH disease progression. Importantly, most models only show simple steatosis and do not lead to NASH or fibrosis. A recently characterized Western diet plus sugar water (WDSW)-induced mouse model of NAFLD with a mixed background, C57Bl/6J and 129S1/SvlmJ (B6/129), was shown to recapitulate the key physiological, pathological, metabolic, histological, and cellular signaling changes seen in human NASH patients [11,12]. This model represents the best available preclinical model of NASH.

In this study, we examined the effect of BBR on NASH disease progression using this WDSW-induced NAFLD mouse model and fully characterized the gene expression, signaling pathways, and histological and metabolic changes. The results indicated that BBR is a promising therapeutic agent for NASH by modulating multiple pathways.

## 2. Materials and Methods

### 2.1. Reagents

Berberine chloride hydrate (BBR) was purchased from Sigma (St. Louis, MO, USA, Cat #14050). Oil Red O and common laboratory chemicals were purchased from Sigma Aldrich (St. Louis, MO, USA). All antibodies used in this study are listed in Table S6.

### 2.2. Mice

A mouse strain with a mixed background, C57Bl/6J and 129S1/SvlmJ (B6/129), was originally obtained from Dr. Sandra Erickson (UCSF, San Francisco, VA, USA) as a gift. The strain was bred at the animal facility of McGuire VA Medical Center by F2 intercross. After more than 20 generations of brother–sister mating, these mice develop classic steatosis in 4–8 weeks and progression to NASH and fibrosis in 16–24 weeks and spontaneous hepatocellular carcinoma (HCC) in 52 weeks on a high-fat and high-carbohydrate diet (Western diet, WD, Harlan, TD88137, Table S5) with a high-fructose/glucose solution (SW, 23.1 g/L d-fructose plus 18.9 g/L d-glucose) [11]. Both male and female mice at the ages of 8–12 weeks were used in this study. Mice were randomly assigned to the normal diet control group (ND), NASH group, and NASH + BBR group (*n* = 10). ND control mice were fed a standard chow diet (CD, Harlan TD 7012) with normal water (NW). NASH and NASH + BBR mice were fed WD plus SW (WDSW) for 21 weeks. At the 12 week feeding time point, NASH + BBR mice were treated with BBR (50 mg/kg) daily via oral gavage and NASH mice were given the same volume of the vehicle for 9 weeks. Mice were weighed twice a week and the dosage of BBR was adjusted on the basis of body weight. All mice were housed in a 12 h light/12 h dark cycle with a controlled room temperature between 21 and 23 °C and free access to water. All the experimental procedures were performed according to protocols approved by the McGuire VA Medical Center and Virginia Commonwealth University Institutional Animal Care and Use Committee. All animal experiments were performed in accordance with institutional guidelines for ethical animal studies. At the end of the experiment, mice were weighed and anesthetized by exposure to inhaled isoflurane. The blood was collected by cardiac puncture. The serum was collected and stored at −80 °C for later analysis. After euthanasia, the liver was collected for histological analysis, RNA profiling, and Western blot analysis.

### 2.3. RNA Sequencing (RNAseq) and Bioinformatic Analysis

Total liver RNA was isolated using Chemagic Prepito^®^-D Nucleic Acid Extractor (PerkinElmer, Waltham, MA, USA) with a Prepito RNA kit (PerkinElmer, USA). The RNAseq with ribosomal RNA (rRNA) depletion was done by Genewiz Company using the Illumina Hiseq^®^ X platform (Genewiz Co., South Plainfield, NJ, USA).

Sequencing reads were trimmed and filtered using bbduk to remove adapters and low-quality reads [13]. Reads from mouse samples were mapped to Ensembl GRCm38 transcripts annotation (release 82), using RSEM [14]. Gene expression data normalization and differential expression analysis were performed using the R package edgeR [15]. Significantly up- or downregulated genes were determined as fold change ≥2 and q-value < 0.05. Hierarchical clustering was performed to show distinguishable mRNA expression profiles among the samples. The volcano graph and heatmaps were created to visualize significantly dysregulated mRNAs using GraphPad Prism (version 8; GraphPad Software Inc., San Diego, CA, USA). Gene Ontology (GO) analysis was used to investigate three functionality domains: biological process (BP), cellular component (CC), and molecular function (MF) using DAVID (Database for Annotation, Visualization, and Integrated Discovery) v6.8 (https://david.ncifcrf.gov/). Pathway analysis was performed to functionally analyze and map genes to Kyoto Encyclopedia of Genes and Genomes (KEGG) pathways (https://pathview.uncc.edu/).

### 2.4. Serum and Liver Biochemical Analysis

The serum levels of alkaline phosphatase (ALP), aspartate aminotransferase (AST) and alanine aminotransferase (ALT), total triglyceride (TG), total cholesterol (TC), very-low-density lipoprotein (VLDL), and albumin (ALB) were determined using the Alfa Wassermann Vet ACE Axcel^®^ System with commercially available assay kits (Alfa Wassermann diagnostic technologies, NJ, USA). Liver tissues were homogenized with a 10-fold 5% NP-40/ddH_2_O solution. The homogenates then were heated to 80–100 °C in a water bath for 2–5 min or until the NP-40 solution became cloudy. After cooling down to room temperature, the homogenates were centrifuged for 2 min at 16,000 rpm. The TG and TC levels in the supernatant of the liver homogenates were analyzed by using the same method as described above and normalized using total protein concentration [16].

### 2.5. Histological Analysis and Immunohistochemical Staining

Liver tissues were processed for hematoxylin and eosin (H&E) stains and immunohistochemistry (IHC) staining for CK-19, MPO, and F4/80 at the Cancer Mouse Models Core at the VCU Massey Cancer Center (Richmond, VA, USA). Picro Sirius Red Staining was performed using the Picro Sirius Red Stain Kit (Abcam, USA) with the paraffin-embedded tissue sections according to the manufacturer’s instructions. Frozen liver tissue sections (8 μm in thickness) were preserved in 3.7% formaldehyde for 10 min and followed by Oil Red O staining described previously [17]. All the stained slides were scanned using a Vectra Polaris Automated Quantitative Pathology Imaging System (Akoya Biosciences, MA, USA) and the images were captured using Phenochart software (Akoya Biosciences, MA, USA).

The H&E slides were analyzed blindly by two clinical pathologists using the NASH score system. For each liver slide, macrovesicular steatosis, hepatocellular ballooning, and lobular inflammation were graded using the NASH score system. Steatosis was graded on a scale of 0 to 3 (0: <5%; 1:5–33%; 2: 34–66%; 3: >67%). Ballooning hepatocytes were graded as 0 (none), 1 (when few hepatocytes presented a round-shaped, reticulated, and pale cytoplasm, but with normal dimensions), and 2 (when there was a cluster of prominent ballooning hepatocytes). The presence of inflammatory foci within the lobule or within the sinusoids was graded as 0 (none), 1 (<2 foci per 20× field), 2 (2–4 foci per 20× field), and 3 (>4 foci per 20× field). The NAFLD activity score (NAS) was calculated by the addition of grades of steatosis, inflammation, and ballooning. Detection of collagen fibers was performed by Picro Sirius Red Staining and was scored according to the NASH-CRN system [18].

### 2.6. Bile Acid (BA) Analysis

The serum and liver tissues were processed for bile acid analysis, as described previously [19]. The processed samples were filtered using Whatman^TM^ Mini-UniPrepTM syringeless filters (0.2 µm PTFE, Cat. #09-923-102). High-throughput profiling of BAs was performed using a Shimadzu liquid chromatography/mass spectrometry (LC–MS) 8600 system. BA metabolite data in the original scale in terms of raw area counts were normalized by median centering. Missing values were imputed with the lower limit of detection for a given metabolite. When ≥30% of the values were missing, data were excluded from analyses. The peak and area under the curve for individual analytes were related to validated library standards to compute BA levels, which are read out as related to these library standards.

The bile acids, including deuterium (d4)-labeled internal standards (IS), used in this study, are listed in Table S7**.** GβMCA, TβMCA, ωMCA, TωMCA, GHCA, THCA, GHDCA, *d_4_*-GCA, *d_4_*-TCA, *d_4_*-CDCA, *d_4_*-GDCA, *d_4_*-DCA, *d_4_*-LCA were purchased from Cayman Chemical Co. (Ann Arbor, MI). αMCA, TαMCA, βMCA, HCA, THDCA, MDCA, GLCA, TLCA, isoDCA, isoLCA, 7-KetoDCA, 7-KetoLCA, 12-KetoLCA, Allo-isoLCA, and DhLCA were available from Steraloids Inc (Newport, RI). All other bile acid standards were obtained from Sigma-Aldrich (St Louis, MO). LC–MS-grade solvents and chemicals were purchased from Fisher Scientific (New Lawn, NJ), unless indicated otherwise. The stock solutions of the bile acid standards at a concentration of 100 μM (each bile acid) were prepared in 70% ethanol. Standard bile acid mixtures for calibration curves were prepared at the concentrations of 0.01, 0.03, 0.1, 0.3, 1, 3, 10, and 30 µM in acetonitrile/methanol/water (25:25:50, *v*/*v*). IS solution, which was a mixture of 10 stable isotope-labeled bile acids (*d*_4_-CA, *d*_4_-GCA, *d*_4_-TCA, *d_4_*-CDCA, *d_4_*-DCA, *d*_4_-GCDCA, *d*_4_-TCDCA, *d_4_*-LCA, *d_4_*-GLCA, *d_4_*-TLCA), was also prepared at 0.5 µM (each bile acid) in acetonitrile/methanol/water (25/25/50, *v*/*v*/*v*). To construct the calibration curves, 10 μL of each standard solution was mixed with 20 μL of IS solution and diluted to 400 µL with acetonitrile/methanol/water (25/25/50, *v*/*v*/*v*). A 2 μL aliquot was injected into the LC/MS/MS system.

For quantification of bile acids in the liver, 20 mg of liver tissue was homogenized with 500 µL of acetonitrile/methanol/water (25/25/50, *v*/*v*/*v*) using the Precellys Evolution Tissue Homogenizer with Cryolys (Bertin Corp, Rockville, MD). After centrifugation at 12,000× *g* for 2 min at room temperature, the supernatant (10 µL) was spiked with IS solution (20 µL, 10 pmol). The mixture was diluted to 400 µL with methanol/acetonitrile/water (25/25/50, *v*/*v*/*v*). For the serum specimen, 10 µL was spiked with IS solution (20 µL, 10 pmol), and the mixture was diluted with 250 µl of acetonitrile/methanol (1:1, *v*/*v*). After centrifugation at 12,000× *g* for 2 min at room temperature, the supernatant (200 µL) was diluted with water (200 µL). All samples were filtered through 0.2 µm PTFE membrane, and 2 μL aliquots were injected into the LC/MS/MS system. The Shimadzu LCMS-8600 CL liquid chromatography triple-quadrupole tandem mass spectrometer equipped with a dual ion source (DUIS) interface was used. Data were collected and processed using Lab Solutions software. Table S8 lists the multiple reaction monitoring (MRM) transitions, collision energy (CE), and retention time (RT) data for the measured bile acids.

### 2.7. Quantitative RT-PCR

Total liver RNA was isolated using Chemagic Prepito^®^-D Nucleic Acid Extractor (PerkinElmer, Waltham, MA, USA) with a Prepito RNA kit (PerkinElmer, USA). The complementary DNA (cDNA) synthesis and quantitative RT-PCR analysis of relative mRNA expression levels of target genes were performed as previously described [16]. The primer sequences can be provided upon request.

### 2.8. Immunoblotting Analysis

Total proteins were prepared using cold RIPA buffer. Nuclear and cytosolic proteins were isolated, as previously described [20]. Protein concentration was measured using the Bio-Rad Protein Assay reagent. Proteins were resolved on 10% SDS-PAGE and transferred to nitrocellulose membranes (Thermo, Waltham, MA, USA). The target proteins were probed with the specific primary antibodies and detected using HRP-conjugated secondary antibodies and ECL reagents (Thermo, USA). Images were captured using the Bio-Rad Gel Doc XR+ imaging system (Hercules, CA, USA). The density of immunoblotted bands was analyzed using BioRad Image Lab computer software and normalized with β-actin or histone 3.

### 2.9. Statistical Analysis

Data are expressed as the mean ± SEM from at least three independent experiments. One-way analysis of variance (ANOVA) followed by Tukey’s post hoc test was performed to analyze the differences among multiple groups by GraphPad Prism (version 8; GraphPad Software Inc., San Diego, CA). Student’s *t*-test was used to analyze the difference between the two groups. A *p*-value <0.05 was considered statistically significant.

## 3. Results

### 3.1. BBR Significantly Prevented NAFL to NASH Progression in WDSW-Fed Mice

**Figure 1:**
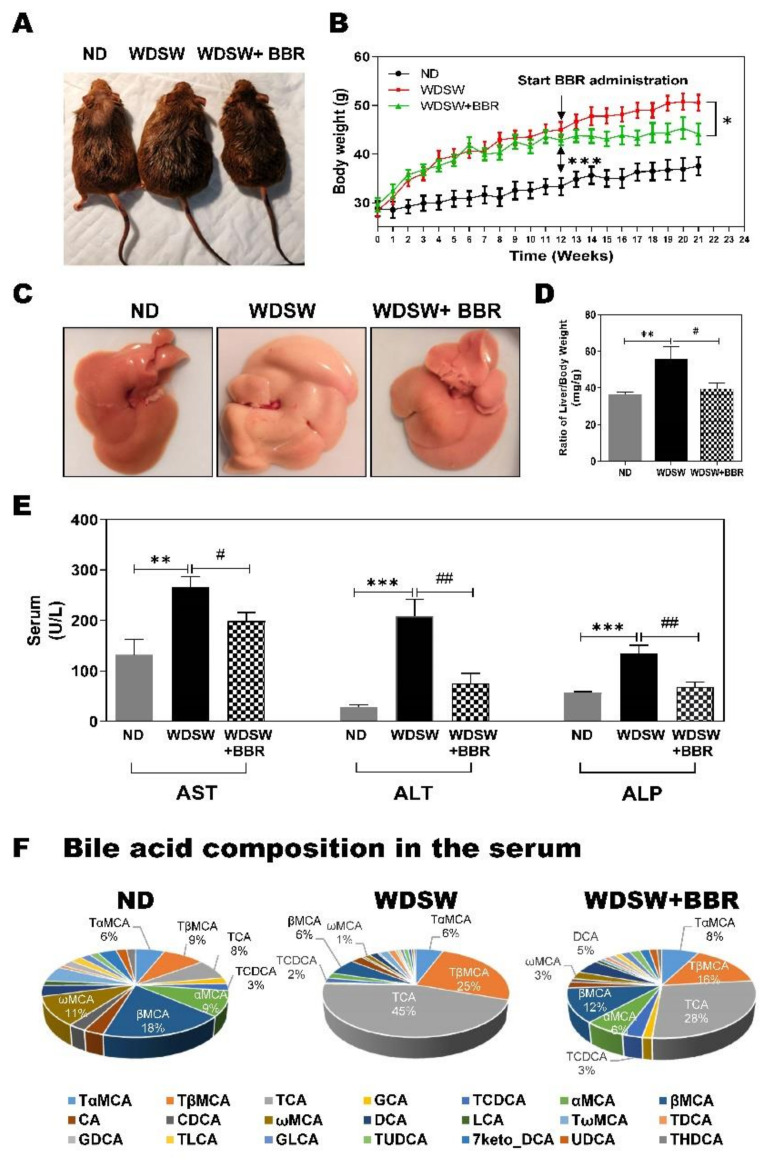
Effect of berberine (BBR) on biometric parameters, serum biochemical parameters, and bile acid profile in the Western diet plus sugar water (WDSW)-induced nonalcoholic fatty liver disease (NAFLD) mouse model. The F2 B6/129 mice were fed a normal chow diet with tap water (ND) or Western Diet with high fructose/glucose (WDSW) for 12 weeks. WDSW animals were treated with vehicle (*n* = 10) or BBR (50 mg/kg/day, *n* = 11) via oral gavage once daily for 9 weeks while continuing feeding with WDSW. ND mice (*n* = 9) did not receive any treatment. (**A**) Representative image of whole mice in each group. (**B**) Bodyweight change during the experimental feeding period of 21 weeks. (**C**) The representative image of the liver. (**D**) The ratio of liver to body weight. (**E**) Liver functional enzyme analysis. (**F**) Bile acid composition profile in the serum expressed by % of total bile acids. Data are expressed as the mean ± standard error of the mean (SEM). Statistical significance: * *p* < 0.05 vs. ND, ** *p* < 0.01 vs. ND, *** *p* < 0.001 vs. ND; ^#^ *p* < 0.05 vs. WDSW, ^##^ *p* < 0.01 vs. WDSW. Abbreviations: ALP, alkaline phosphatase; AST, aspartate aminotransferase; ALT, alanine aminotransferase.

To examine the effect of BBR on NASH disease progression, the F2 generation of the mixed-background C57Bl/6J and 129S1/SvlmJ (B6/129) mice were first fed a WDSW for 12 weeks to induce steatosis (NAFL) followed by treatment with BBR (50 mg/kg) or vehicle control for an additional 9 weeks with continuous feeding with WDSW. The control mice were fed ND and regular water. As shown in Figure 1A,B, WDSW feeding significantly increased body weight after 12 weeks compared to the ND control. Continuous feeding with WDSW further increased body weight, which was significantly reduced by BBR treatment. In order to determine whether BBR-induced body weight loss was caused by less food intake, the food intake of the mice in WDSW and WDSW + BBR groups was monitored. As shown in Figure S1A (Supplementary Materials), the average daily food intake of mice in WDSW and WDSW + BBR is similar. The feeding with WDSW dramatically increased liver size with a much lighter color compared to the ND control, which was reduced by BBR treatment (Figure 1C,D). WDSW feeding also significantly increased serum aspartate aminotransferase (AST), alanine aminotransferase (ALT), and alkaline phosphatase (ALP) levels, which were reduced by BBR treatment (Figure 1E). Furthermore, WDSW feeding significantly increased total serum cholesterol (TC) and glucose levels and decreased serum triglycerol (TG) and very-low-density lipoprotein (VLDL) levels. BBR treatment reduced serum TC and glucose levels but did not affect TG and VLDL levels. The total bilirubin and albumin levels remained unchanged (Figure S1B,C, Supplementary Materials).

We recently reported that NASH disease severity is associated with specific changes in circulating bile acids in human patients [21]. To examine whether the serum bile acid profile of this mouse model has similar changes as seen in NASH patients, we measured more than 30 different bile acids in the serum and liver using LC–MS. As shown in Figure S1D (Supplementary Materials) and Figure 1F, WDSW feeding not only increased the total serum bile acid level but also dramatically changed the ratios of primary bile acids to secondary bile acids and conjugated bile acids to unconjugated bile acids. The most obvious change was the significant increase of taurocholic acid (TCA) level in the serum (Figure S1D). The percentage of TCA in total bile acids was increased from 8% (ND) to 45% (WDSW) after a total of 21 weeks of WDSW feeding (Figure 1F). However, WDSW-induced changes of serum bile acids were markedly inhibited by BBR treatment. BBR not only reversed the WDSW-induced increase in total bile acid levels and TCA level but also significantly reduced the impact of WDSW on bile acid composition. The percentage of the TCA level was reduced from 45% to 28%. In addition, the WDSW-induced increase in the percentage of tauro-β-muricholic acid (TβMCA) (from 9% to 25%) was reduced by BBR to 16% (Figure 1F; Table S1). The total conjugated bile acids and the ratio of total primary bile acids to secondary bile acids were also increased by WDSW feeding, which was reduced by BBR (Table S2).

**Figure 2:**
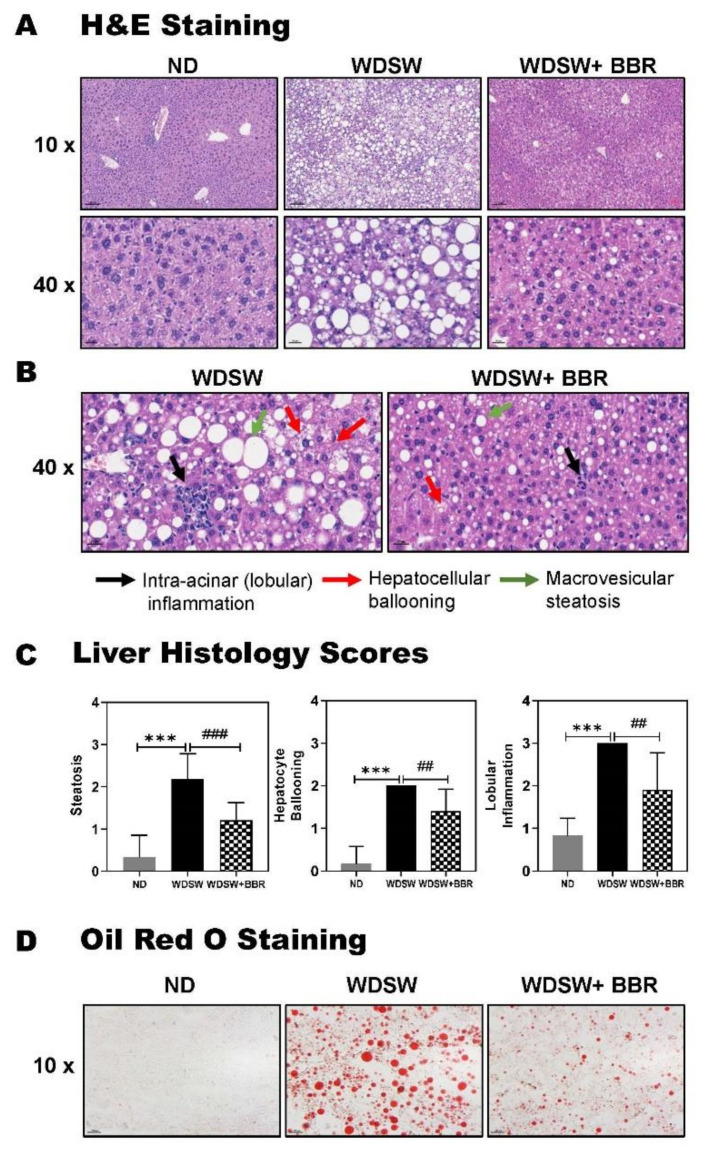
Effect of BBR on nonalcoholic steatohepatitis (NASH) progression in the WDSW-induced NAFLD mouse model. (**A**) Representative images of hematoxylin and eosin (H&E) staining of the liver slides (scale bar, 100 μm for 10×, 20 μm for 40× magnification). (**B**) Representative images of intra-acinar (lobular) inflammation, hepatocellular ballooning, and macrovesicular steatosis of H&E-stained liver slides (scale bar, 20 μm for 40× magnification). (**C**) Liver histology scores, including steatosis, hepatocellular ballooning, and lobular inflammation. Data are expressed as the mean ± SEM. Statistical significance: *** *p* < 0.001 vs. ND; ^##^ *p* < 0.01 vs. WDSW, ^###^ *p* < 0.001 vs. WDSW. (**D**) Representative images of liver sections stained with Oil red O (scale bar, 100 μm for 10× magnification).

Histological analysis of the liver tissue indicated that WDSW-feeding induced NASH after 5 months. As shown in Figure 2A–C and Figure S2, hematoxylin and eosin (H&E) staining showed that WDSW-feeding induced NASH with severe steatosis, lobular inflammation, and hepatocellular ballooning, which were markedly reduced by BBR. Oil Red O staining further showed that BBR significantly reduced WDSW-induced hepatic lipid accumulation (Figure 2D).

### 3.2. BBR Prevented NASH Progression by Modulating Global Transcriptomic Profile in WDSW-Fed Mice

**Figure 3:**
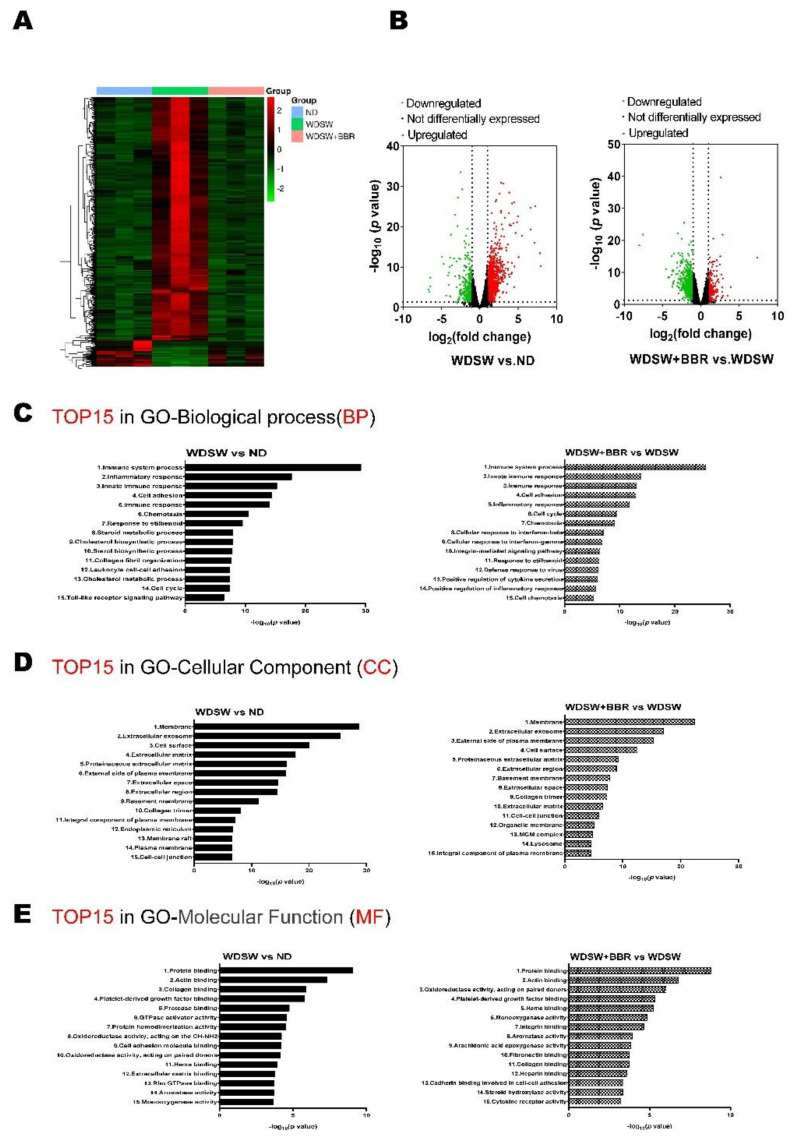
Heatmap, volcano plot, and Gene Ontology (GO) for differentially expressed genes (DEGs) in liver tissues of the two comparisons: WDSW vs. ND and WDSW + BBR vs. WDSW. Total liver RNA from triplicate samples in each experimental group was processed for transcriptome sequencing (RNAseq). Differentially expressed genes (DEGs) between the two groups were identified using fold change (FC) and *p*-values (FC ≥2 and *p*-value <0.05). (**A**) Hierarchical clustering heatmaps for DEGs in both WDSW vs. ND and WDSW + BBR vs. WDSW groups. A *Z*-score was calculated for the RNAseq data to normalize tag counts. Red and blue colors indicate high and low gene expression, respectively. (**B**) Volcano plots of the two comparisons: WDSW vs. ND and WDSW + BBR vs. WDSW. Red dots indicate upregulated genes; green dots indicate downregulated genes; black dots indicate not differentially expressed genes. Top 15 enriched terms of the DEGs in GO-BP (biological process) (**C**), GO-CC (cellular component) (**D**), and GO-MF (molecular function) (**E**) of the two comparisons: WDSW vs. ND and WDSW + BBR vs. WDSW.

To delineate the underlying mechanisms via which BBR prevents NASH disease progression, we performed RNAseq transcriptome analysis. As shown in Figure S3 (Supplementary Materials), WDSW feeding induced upregulation of 1035 genes and downregulation of 284 genes, while BBR treatment downregulated 770 genes and upregulated 184 genes, which were induced by WDSW feeding. The heatmap shown in Figure 3A displays the distinct differences between the ND and the WDSW feeding group, while the BBR treatment group showed a similar profile to the ND group. The volcano plot further showed that the WDSW-induced change in gene expression was significantly reversed by BBR (Figure 3B). Gene Ontology analysis showed that WDSW feeding significantly impacted the major pathways in biological process, cellular component, and molecular function related to metabolic processes and immunological responses (Figure 3C–E). BBR was able to target the majority of the biological processes (eight out of 15), cellular components (11 out of 15), and molecular functions (eight out of 15) affected by WDSW feeding, such as immune system process, inflammation, cell adhesion, extracellular matrix, cell–cell junction, chemotaxis, and protein binding.

### 3.3. Effect of BBR on WDSW-Induced Dysregulation of Fatty Acid and Lipid Metabolism

**Figure 4:**
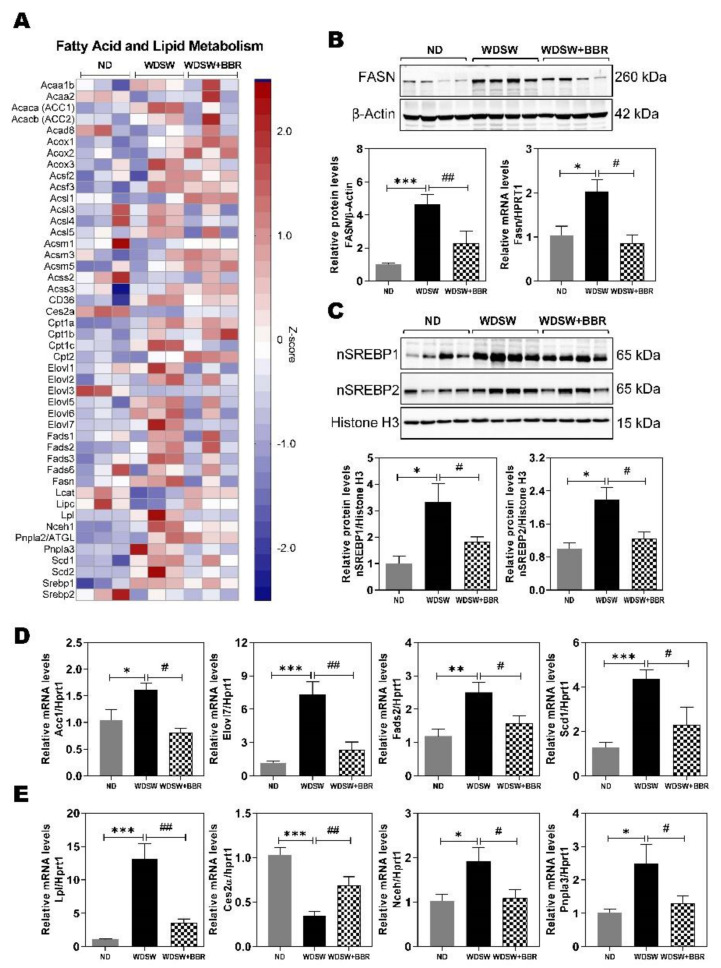
Effect of BBR on NASH-associated dysregulation of fatty acid and lipid metabolism. (**A**) Representative heatmap of the key genes involved in fatty acid and lipid metabolism. A *Z*-score was calculated for the RNAseq data to normalize tag counts. Red and blue colors indicate high and low gene expression, respectively. (**B**) Representative image of the Western blot of fatty acid synthase (Fasn), used as an internal control. (**C**) Representative immunoblot images of nuclear sterol regulatory element-binding protein 1 (Srebp1) and Srebp2 are shown and normalized with histone H3 as an internal control. (**D**,**E**) Relative messenger RNA (mRNA) levels of the key genes involved in fatty acid and lipid metabolism were determined by real-time RT-PCR and normalized with HPRT1(Hypoxanthine Phosphoribosyltransferase 1) as an internal control. (**D**) Genes involved in fatty acid synthesis: acetyl CoA carboxylase (Acc1), elongation of very-long-chain fatty acids member 7 (Elovl7), fatty acid desaturase 2 (Fads2), stearoyl-coenzyme A desaturase 1 (Scd1). (**E**) Genes involved in lipid metabolism: carboxylesterase 2A (Ces2α), lipoprotein lipase (Lpl), neutral cholesterol ester hydrolase (Nceh), and patatin-like phospholipase domain containing 3 (Pnpla3). Data are expressed as the mean ± SEM. Statistical significance: * *p* < 0.05 vs. ND, ** *p* < 0.01 vs. ND, *** *p* < 0.001 vs. ND; ^#^ *p* < 0.05 vs. WDSW, ^##^ *p* < 0.01 vs. WDSW.

One of the major characteristics during the development of NAFL/NASH is the dysregulation of lipid metabolism. Consistent with the previous studies, these mice developed NASH in 20 weeks. The de novo lipogenesis pathway was persistently activated. As shown in Figure S4 (Supplementary Materials), WDSW feeding upregulated the majority of the genes involved in the fatty acid biosynthesis pathway, while BBR treatment reversed its effect. The heatmap shown in Figure 4A indicated that the WDSW feeding-induced changes in gene expression in fatty acid and lipid metabolism were inhibited by BBR, such as fatty acid synthase (Fasn), acetyl CoA carboxylase (Acc1), long-chain fatty acid CoA ligase 5 (Acsl5), and elongation of very-long-chain fatty acids members 5, 6, and 7 (Elovl5, 6, and 7), fatty acid desaturases (Fads1, 2, and 3), stearoyl-coenzyme A desaturase 1 (Scd1) and Scd2, carboxylesterase 2A (Ces2α), lecithin cholesterol acyltransferase (Lcat), lipoprotein lipase (Lpl), neutral cholesterol ester hydrolase 1 (Nceh1), and patatin-like phospholipase domain containing 3 (Pnpla3). Western blot analysis and real-time PCR further confirmed that WDSW-induced upregulation of Fasn was significantly inhibited by BBR (Figure 4B). Although BBR did not affect the messenger RNA (mRNA) expression levels of sterol regulatory element-binding protein 1 and 2 (Srebp1 and 2), the master regulators of hepatic lipid metabolism, WDSW-induced activation of Srebp1 and 2 was reduced by BBR as indicated by reduced protein levels of the nuclear forms of Srebp1 and 2 (Figure 4C). We further confirmed the expression of several key genes involved in hepatic lipid metabolism by real-time RT-PCR. As shown in Figure 4D,E, WDSW-induced upregulation of the mRNA expression levels of Acc1, Eovl7, Fads2, Scd1, Lpl, Nceh1, and Pnpla3 and downregulation of the mRNA level of Ces2α were reversed by BBR.

### 3.4. Effect of BBR on WDSW-Induced Inflammation and Oxidative Stress

**Figure 5:**
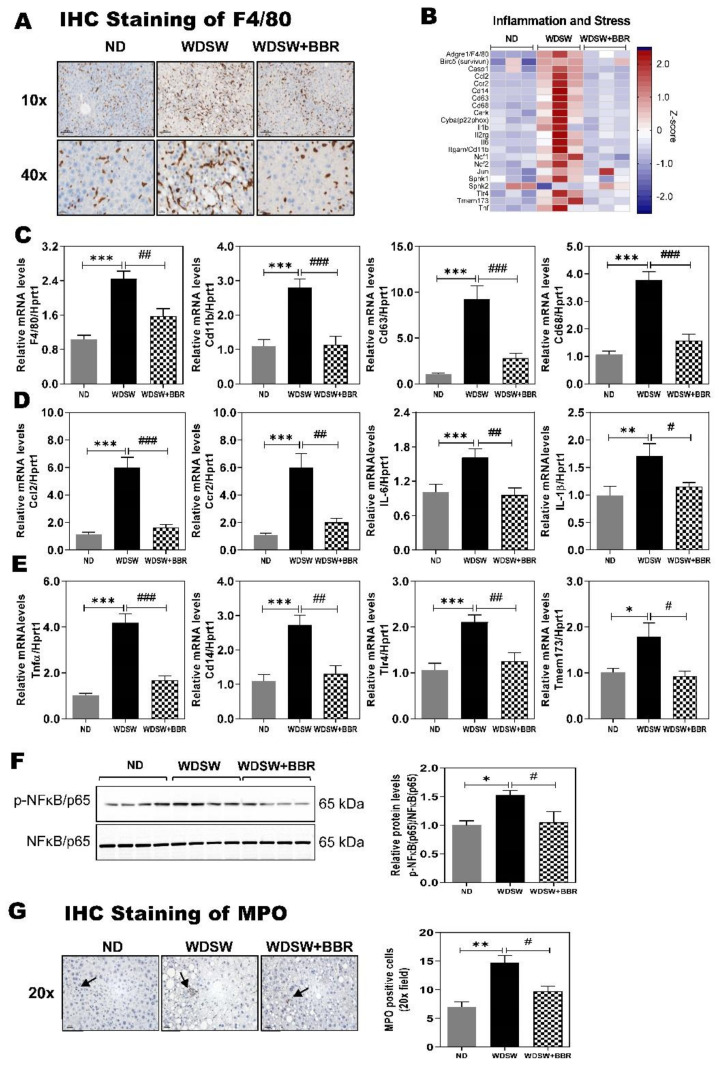
Effect of BBR on NASH-associated inflammation and stress responses. (**A**) Representative images of immunohistochemistry (IHC) staining of F4/80 (scale bar, 100 μm for 20× and 20 μm for 40× magnification). (**B**) Representative image of a heatmap of the key genes involved in inflammation and stress response. A *Z*-score was calculated for the RNAseq data to normalize tag counts. Red and blue colors indicate high and low gene expression, respectively. (**C**–**E**) Relative mRNA levels of genes involved in inflammation and stress associated with NASH were determined by real-time RT-PCR and normalized with HPRT1 as an internal control. (**C**) Macrophage markers: Cd11b, Cd63, and Cd68. (**D**) Chemokines and interleukin family of cytokines: chemokine (C–C motif) ligand 2 (Ccl2), chemokine (C–C motif) receptor 2 (Ccr2), interleukin (IL)-6, and IL-1β. (**E**) Innate immune response inflammatory markers: tumor necrosis factor alpha (Tnfα), Cd14, Toll-like receptor 4 (TLR4), and transmembrane protein 173 (TMEM173). Abbreviation: NC, negative control. (**F**) Representative immunoblot images of phosphorylated (p)-nuclear factor (NF)-κB/p65 and NF-κB/p65 and relative protein levels. (**G**) Representative images of IHC staining of myeloperoxidase (MPO) (scale bar, 100 μm for 20×) and the number of MPO positive cells. Data are expressed as the mean ± SEM. Statistical significance: * *p* < 0.05 vs. ND, ** *p* < 0.01 vs. ND, *** *p* < 0.001 vs. ND; ^#^ *p* < 0.05 vs. WDSW, ^##^ *p* < 0.01 vs. WDSW; ^###^ *p* < 0.001 vs. WDSW.

Our recent study and studies from others have shown that BBR is a potent anti-inflammatory and antioxidative agent [22,23,24]. Inflammation and oxidative stress response are the key drivers for NASH disease progression [25]. As shown in Figure 5A, WDSW feeding resulted in the infiltration of macrophages to the liver as indicated by the immunohistochemical (IHC) staining of F4/80 antigen, a mature cell surface glycoprotein expressed at high levels on various macrophages. BBR treatment significantly reduced the F4/80 positive cells in the liver. RNAseq analysis further showed that WDSW feeding markedly induced activation of the inflammatory and stress response, which were inhibited by BBR (Figure S5A). Consistent with the IHC staining, RNAseq data also showed that the mRNA level of F4/80 was significantly upregulated in WDSW-fed mice and reversed by BBR treatment (Figure 5B). WDSW feeding also significantly increased the mRNA expression levels of the cell surface adhesion molecules, inflammatory cytokines, chemokines, cell surface antigens, Toll-like receptors (TLRs), and genes related to cell apoptosis, such as integrin alpha M (also known as Cd11b), interleukin 6 (IL-6), IL-1β, tumor necrosis factor α (Tnfα), chemokine (C–C motif) ligand 2 (Ccl2, also known as monocyte chemoattractant protein 1 (Mcp1)), chemokine (C–C motif) receptor 2 (Ccr2), Cd63, Cd68, Cd14, transmembrane protein 173 (TMEM173, also known as stimulator of interferon genes (STING)), TLR4, Jun (jun proto-oncogene), ceramide kinase (Cerk), baculoviral inhibition of apoptosis protein repeat-containing 5 (Birc5, also known as survivin), caspase 1, etc. The inhibitory effect of BBR on WDSW feeding-induced upregulation of major genes involved in inflammatory and stress response was further confirmed by real-time RT-PCR (Figure 5C–E; Figure S5B). We further confirmed the activation of nuclear factor (NF)-κB by Western blot analysis. As shown in Figure 5F, the WDSW-induced increase in p-NFκB/p65 was inhibited by BBR.

Neutrophils are the most abundant leukocytes in circulation and play a crucial role in host innate immune responses during infection. However, improper activation and infiltration of neutrophils have been linked to tissue damage in the different disease settings, including various liver diseases [7,26]. The ratio of neutrophil to lymphocyte has been identified as a better predictor of NASH fibrosis [27]. The neutrophil-mediated oxidative burst is the major contributor to reactive oxygen species (ROS)-mediated liver damage in NAFLD. The IHC staining of myeloperoxidase (MPO) indicated neutrophil infiltration in the liver (Figure 5G). Our RNAseq data showed that in this NAFLD mouse model, the expression levels of key genes involved in neutrophil activation were significantly upregulated, which were markedly inhibited by BBR treatment (Figure S6A). We further confirmed the expression levels of NADPH (Nicotinamide adenine dinucleotide phosphate) oxidase 2 (Nox2, also known as neutrophil cytochrome b heavy chain (Cybβ), or p91phox), the major components of Nox2 complex, including neutrophil cytosolic factor 1 (Ncf1, also known as p47phox), Ncf2 (also known as p67phox), Ncf4 (also known as p40phox), and Cybα (also known as p22phox), as well as IL-2 receptor gamma unit (IL2rg), elastin, selectin, and vascular cell adhesion molecule 1 (Vcam1), by real-time RT-PCR. As shown in Figure S6B (Supplementary Materials), BBR treatment blocked WDSW-feeding-induced upregulations of these genes, except for elastin. Pathway analysis further showed that oxidative phosphorylation in mitochondria was significantly impaired by WDSW feeding, and BBR was able to counteract the effect of WDSW on multiple components (Figure S7).

### 3.5. Effect of BBR on WDSW-Induced Dysregulation of Hepatic Bile Acid Metabolism

**Figure 6:**
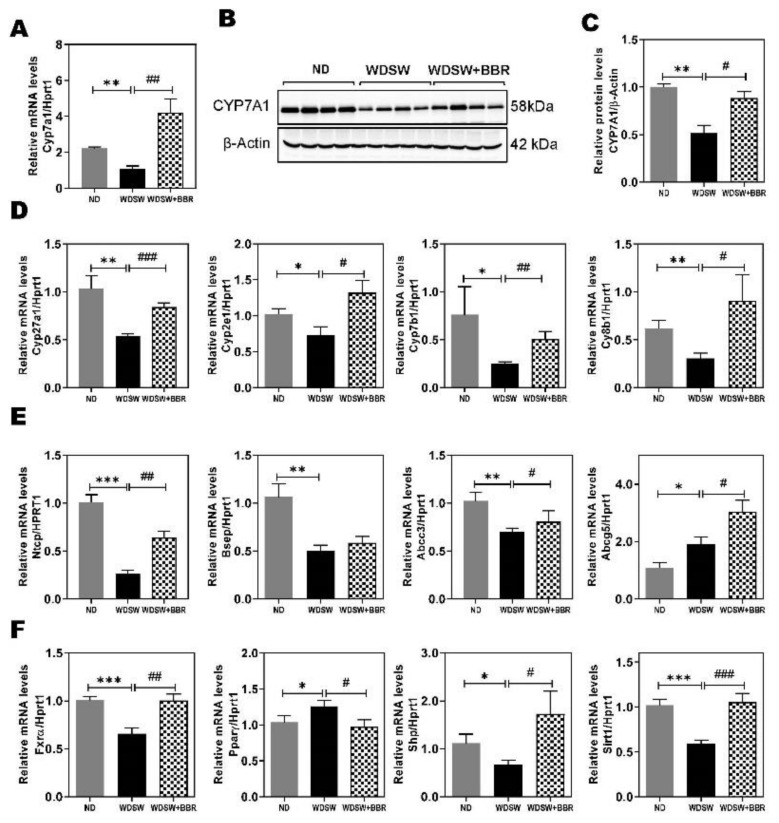
Effect of BBR on NASH-associated dysregulation of bile acid metabolism. (**A**) Relative mRNA level of Cyp7α1(Cholesterol 7 alpha-hydroxylase). (**B**) Representative immunoblot images of Cyp7α1 and β-actin are shown (**C**) Relative protein level of Cyp7α1. (**D**) Relative mRNA levels of key enzymes in hepatic bile acid synthesis: Cyp27a1, Cyp2e1, Cyp7b1, and Cyp8b1. (**E**) Relative mRNA levels of hepatic bile acid transporters: Ntcp (sodium/bile acid cotransporter), Bsep (bile salt export protein), Abcc3(ATP binding cassette subfamily C member 3) and Abcg5(ATP binding cassette subfamily G member 5). (**F**) Relative mRNA levels of Fxrα (farnesoid X receptor α), Shp(short heterodimer partner), Pparγ(Peroxisome proliferator-activated receptor gamma), and Sirt1(Sirtuin 1). Both β-actin and HPRT1 were used as internal controls. Data are expressed as the mean ± SEM. Statistical significance: * *p* < 0.05 vs. ND, ** *p* < 0.01 vs. ND, *** *p* < 0.001 vs. ND; ^#^ *p* < 0.05 vs. WDSW, ^##^ *p* < 0.01 vs. WDSW; ^###^ *p* < 0.001 vs. WDSW.

Bile acids are important signaling molecules involved in the regulation of hepatic lipid, glucose, and energy metabolism [28]. We also showed that BBR was able to prevent WDSW-induced upregulation of circulation bile acids and disruption of bile acid homeostasis (Figure 1F; Figure S1D and Table S1, Supplementary). In our NAFLD mouse model, the RNAseq data displayed a dramatic downregulation of numerous genes in primary bile acid biosynthesis (Figure S8A), including the two rate-limiting enzymes for bile acid synthesis, Cyp7a1(Cholesterol 7 alpha-hydroxylase) and Cyp27a1. BBR treatment markedly upregulated most of the genes in bile acid biosynthesis (Figure S8B). WDSW feeding also induced changes in major hepatic transporters (Figure S9), such as sodium/bile acid cotransporter (Ntcp), ATP-binding cassette sub-family B member 11 (Abcb11 or bile salt export protein (Bsep), Abcb1, and Abcb4. The heatmap in Figure S10 (Supplementary Materials) shows that WDSW feeding not only suppressed the expression of many cytochrome P450 (Cyp) family members but also dysregulated hepatic transporters and nuclear receptors. BBR treatment was able to reverse most of the changes induced by WDSW feeding. To further confirm the findings of RNAseq analysis, we first examined the expression of the rate-limiting enzyme of the classical bile acid synthetic pathway, Cyp7a1. As shown in Figure 6A, consistent with RNAseq analysis, real-time RT-PCR showed that BBR markedly upregulated the mRNA expression level of Cyp7a1. The Western blot analysis further showed that WDSW feeding-induced inhibition of Cyp7a1 protein expression was also reversed by BBR (Figure 6B,C). Similarly, the mRNA expression level of Cyp27a1, the rate-limiting enzyme of the alternative pathway of bile acid synthesis, and the expression levels of Cyp2e1, Cyp7b1, and Cyp8b1 were also reduced by WDSW feeding and reversed by BBR treatment (Figure 6D). However, overall, BBR had no significant effect on the WDSW-induced increase of bile acid levels in the liver (Figure S11 and Tables S3 and S4). WDSW feeding also downregulated the expression of Ntcp, Bsep, and Abcc3 but upregulated Abcg5. BBR treatment was able to upregulate Ntcp and Abcc3 but had no effect on Bsep. BBR treatment further increased the WDSW-induced upregulation of Abcg5 (Figure 6E).

Although RNAseq data did not identify significant changes in nuclear receptors, the real-time PCR analysis showed that WDSW feeding significantly reduced farnesoid X receptor α (Fxrα) and short heterodimer partner (Shp) expression and upregulated Peroxisome proliferator-activated receptor gamma (Pparγ) expression, which was reversed by BBR (Figure 6F). Moreover, BBR also prevented WDSW feeding-induced downregulation of the expression of Sirtuin 1 (Sirt1), a key metabolic sensor for regulating lipid and glucose homeostasis [29].

### 3.6. Effect of BBR on WDSW-Induced Hepatic Fibrosis

**Figure 7:**
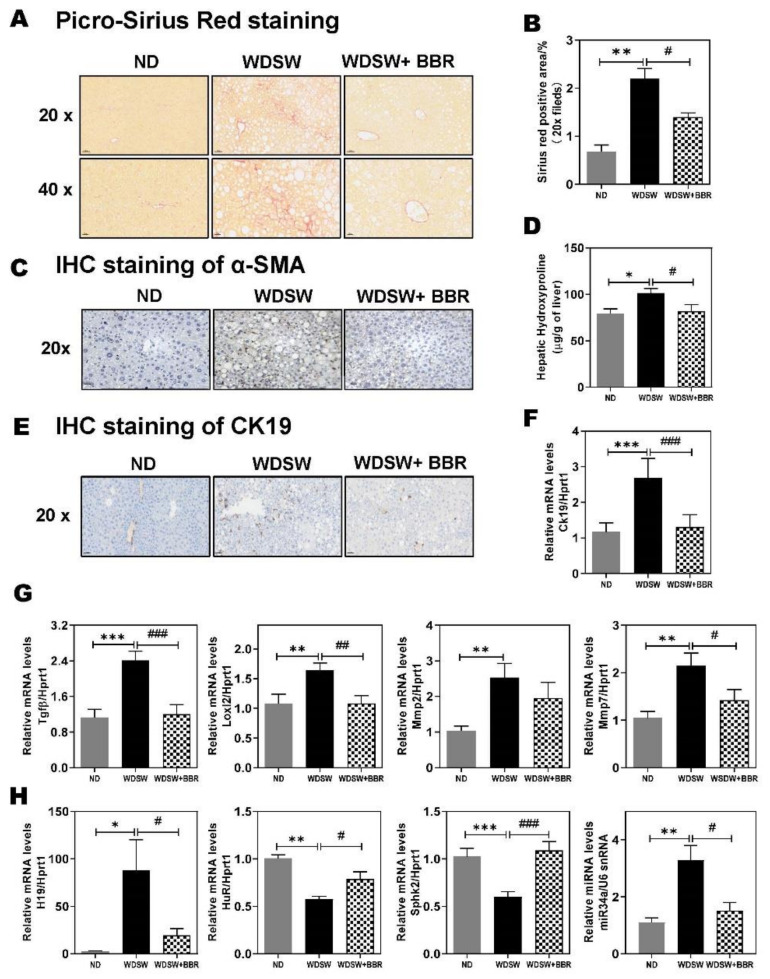
Effect of BBR on NASH-associated hepatic fibrosis (**A**) Representative images of Picro-Sirius Red staining (scale bar, 100 μm for 20× and 20 μm for 40× magnification). (**B**) Quantification of Sirus red staining. (**C**) IHC staining of α-SMA (alpha-smooth actin). (**D**) Hepatic hydroxyproline levels. **(E)** Representative images of IHC staining of CK19(cytokeratin 19). (**F**) Relative mRNA levels of CK19. (**G**) Relative mRNA levels of key genes involved in hepatic fibrosis Tgfβ (transforming growth factor-β), Loxl2 (Lysyl oxidase homolog 2), Mmp2 (matrix metalloproteinase-2), and Mmp7 (matrix metalloproteinase-7). (**H**) Relative expression levels of H19 (long non-coding RNA H19), HuR (human antigen R), and SphK2 (sphingosine kinase 2) were normalized with HPRT1 as an internal control, while the expression levels of microRNA (miR)-34a were normalized with U6 snRNA as an internal control. Data are expressed as the mean ± SEM. Statistical significance: * *p* < 0.05 vs. ND, ** *p* < 0.01 vs. ND, *** *p* < 0.001 vs. ND; ^#^ *p* < 0.05 vs. WDSW, ^##^ *p* < 0.01 vs. WDSW; ^###^ *p* < 0.001 vs. WDSW.

The progression of NAFL to NASH places patients at risk of progression to cirrhosis and hepatocellular carcinoma (HCC). The B6/129 mice develop early fibrosis after 16–20 weeks of WDSW feeding [11]. As shown in Figure S12A (Supplementary Materials), after 21 weeks of WDSW feeding, several fibrotic genes were significantly upregulated, such as collagens (Cols), matrix metallopeptidase (Mmp), transforming growth factor-beta 1(Tgfβ1), Tgfβ receptors (Tgfbrs), connective tissue growth factor (Ctgf), alpha 2, smooth muscle actin (α-Sma), lysyl oxidase-like 2 (Loxl2), long noncoding RNA H19, and thymosin beta 10 (Tmsb10). BBR treatment was able to block the WDSW feeding-induced increase of these fibrotic genes. The Picro-Sirius Red staining of the liver tissue showed that 21 weeks of WDSW feeding induced early fibrosis, which was blocked by BBR treatment (Figure 7A,B). The IHC staining showed BBR significantly reduced the protein level of α-SMA induced by WDSW (Figure 7C). The WDSW feeding-induced increase of hepatic hydroxyproline was also inhibited by BBR (Figure 7D). The cholangiocyte proliferation and ductal reaction also contribute to fibrotic liver injury. IHC staining of keratin 19 (Krt19, also known as CK19) also showed that BBR significantly reduced WDSW-induced cholangiocyte proliferation (Figure 7E). The WDSW-induced upregulation of mRNA levels of Ck19, periostin (Postn), secretin receptor (Sctr), SRY (sex-determining region Y)-box 4 (Sox4), and Sox9 was also blocked by BBR (Figure 7F; Figure S12B). Similarly, BBR was able to inhibit WDSW feeding-induced increase of the mRNA levels of Tgfβ1, Loxl1, Mmp2, and Mmp7 (Figure 7G). Interestingly, WDSW feeding markedly increased expression levels of H19 and microRNA 34a (miR-34a), which were significantly inhibited by BBR (Figure 7H). Furthermore, WDSW feeding downregulated RBP HuR and sphingosine kinase 2 (Sphk2), which was reversed by BBR.

## 4. Discussion

NAFLD/NASH is a metabolic disease, which affects one-quarter of the adult population worldwide. Even though significant advances have been made in the field since the introduction of NAFLD in the clinic in 1986, an effective therapeutic agent is still lacking [1,2]. Lifestyle modifications and exercise are the primary recommendations for NASH patients [30]. Due to the global obesity pandemic, NAFLD is also becoming a major concern in children and adolescents [31,32]. NAFLD and NAFLD-associated metabolic complications impose a substantial financial burden both on society and individuals [33]. Recently, a new terminology, “MAFLD” (metabolic dysfunction associated with fatty liver disease), was suggested by a group of experts to replace NAFLD [34]. The previous proposed “two-hit” hypothesis of NAFLD disease progression is now obsolete, as it is inadequate to explain the heterogeneity of the population with NAFLD. Emerging evidence points toward the “multiple-hits” hypothesis as a more proper explanation of NAFLD pathogenesis [3,35]. In addition to the dysregulation of hepatic lipid metabolism, the progression of NAFL to NASH is linked to individual genetic and epigenetic factors, complex environmental factors, gut microbiome, and the lifestyle of the patients [33].

The beneficial effects of BBR on metabolic and inflammatory diseases have been well documented. The clinical reports and preclinical studies have demonstrated that BBR is a potential antimetabolic disease agent. Although a considerable number of studies have been done to identify the potential underlying mechanisms of BBR’s preventive or therapeutic effect on NAFL/NASH, most of the studies were done either in in vitro cell culture models or in the HFD-feeding animal models, which were unable to recapitulate the NASH pathophysiological process in humans [22,23,36,37,38,39,40,41]. In the current study, we employed histological imaging, molecular biology, biochemical analysis, and RNAseq transcriptome analysis to identify differentially expressed genes followed by GO, KEGG (Kyoto Encyclopedia of Genes and Genomes) pathway, and functional category analysis. The results of this study provide more comprehensive insight into the potential preventive and therapeutic effect of BBR on NASH disease progression using a more clinically relevant NASH mouse model [11,12]. The RNAseq transcriptome analysis strongly indicated that BBR is an ideal candidate for NASH treatment by modulating multiple pathways in the pathogenesis of NASH, such as regulating fatty acid and bile acid metabolism, modulating the innate immune response, inhibiting inflammatory and oxidative stress responses, and modulating the gut microbiome.

Hepatic lipid accumulation is linked to increased uptake or synthesis and decreased oxidation of fatty acids. The results in this study demonstrated that, in these NAFLD mice, WDSW feeding dramatically increased fatty acid synthesis and inhibited fatty acid oxidation (Figure S4; Figure 4). BBR was able to target multiple pathways and components to reduce hepatic lipid accumulation. Both preclinical NAFLD animal models and human clinical studies have shown that inflammation is a major driving force to induce lipid accumulation and NASH progression [11,35,42]. Therefore, anti-inflammation represents a promising therapeutic strategy. It has been well documented in many in vitro and in vivo animal studies that BBR has strong anti-inflammatory activities [37,43,44,45]. Although numerous studies have reported that BBR has a beneficial effect on preventing NAFLD, most of the animal models used were limited to steatosis without significant inflammation and NASH progression. By using RNAseq gene and pathway profiling, we are able to show here that BBR significantly reduced WDSW-induced inflammation and prevented NASH disease progression and early fibrosis. WDSW-induced systemic inflammation was almost blocked by BBR treatment (Figure 2 and Figure 5; Figure S5). BBR not only reduced the inflammatory macrophage infiltration to the liver by decreasing the expression of various cytokines, chemokines, and cell surface adhesion molecules but also markedly reduced neutrophil activation (Figure 5G; Figure S6). The activation of neutrophils is a hallmark of NASH disease progression [46]. The neutrophil extracellular traps are important inducers of oxidative stress and contributors to NASH progression [27,46]. RNAseq data, real-time RT-PCR results, and IHC of MPO indicated that BBR was able to inhibit WDSW-induced activation of neutrophils.

TMEM173 or STING is a major signaling molecule involved in activating the type I interferon-mediated innate immune response. A recent study reported that the expression levels of STING were increased in liver tissues from patients with NAFLD and mice with HFD-induced steatosis [47]. Both RNAseq and real-time PCR results showed that the mRNA level of STING was significantly upregulated in the NASH mouse model, which was completely blocked by BBR (Figure 5B,E). Modulating the innate immune response may represent one of the major molecular mechanisms underlying BBR-mediated anti-inflammatory response in the NASH disease setting. Gene Ontology analysis of RNAseq analysis showed that the immune system process, inflammation, and innate immune response are the top biological processes that changed in the NASH mouse model, which were reversed by BBR (Figure 3C).

Bile acid is exclusively synthesized in the hepatocytes. It is well known that dysregulation of bile acid metabolism contributes to NAFL/NASH disease progression [21,48]. The serum bile acid levels, especially TCA, and the ratio of conjugated to unconjugated primary bile acids were significantly increased in NASH patients, but not NAFL patients [21]. The results in this study indicated that this NAFLD mouse model showed similar changes in circulation bile acid level and composition, as seen in human NASH patients (Figure 1F; Figure S1 and Tables S1 and S2). BBR was able to restore the bile acid homeostasis by modulating the key enzymes of bile acid synthesis, nuclear receptors, and hepatic transporters. Consistent with the previous study, we also found that HFD suppressed hepatic SHP and CYP7A1 expression, which was reversed by BBR treatment (Figure 6) [49]. Since the identification of the first bile acid receptor FXRα in 1999, the role of FXRα in bile acid and lipid metabolism has been extensively studied [50,51,52,53]. Bile acid-induced activation of FXRα plays a critical role in regulating the homeostasis of intrahepatic bile acid circulation [54]. Several FXRα agonists have been developed and are under investigation as the potential therapeutics of NASH [50]. Although BBR increased CYP7A1, the bile acid levels, especially the conjugated bile acids, were significantly reduced (Figure 1F). One potential mechanism is the increased bile acid metabolism. As shown in Figure 6D, HFD-induced downregulation of Cyp7b1 expression was reversed by BBR. Cyp7b1 is a key enzyme involved in the synthesis of oxysterols [55]. However, whether BBR has a direct impact on hepatic and serum oxysterol levels remains to be determined. Our previous studies reported that increased primary conjugated bile acid is responsible for cholestatic liver injury and liver fibrosis by activating sphingosine-1 phosphate receptor 2 and lncRNAH19 [56,57]. Consistently, in the current study, we also found that H19 is dramatically upregulated in the NASH mouse model, markedly inhibited by BBR (Figure 7H). Interestingly, we also found RBP HuR was downregulated by WDSW feeding along with SphK2, which was also inhibited by BBR (Figure 7H). Both HuR and SphK2 were reported to be critical regulators of hepatic lipid metabolism [58,59]. A recent study also reported that miR-34a is a novel serum biomarker for NASH fibrosis [60]. In this study, we also found WDSW-induced upregulation of miR-34a was inhibited by BBR (Figure 7H).

We previously reported that BBR’s beneficial effect on lipid metabolism is closely linked with its ability to modulate gut microbiome and bile acid circulation [10]. Numerous studies have shown that the gut microbiome functions as a hidden organ, which represents an important drugable target for metabolism disease [45]. A recent clinical study reported that BBR’s antidiabetic effect is also associated with its effect on inhibiting DCA biotransformation by *Ruminococcus bromii* [61]. However, it is well characterized that anaerobic bacteria in the genus *Clostridium* play more critical roles in bile acid transformation [62]. In our previous study, BBR showed significant inhibition of the 7α-dehydroxylation conversion of cholic acid (CA) to deoxycholic acid (DCA) [10]. Modulation of gut bile acid metabolism has a profound impact on systemic metabolism by regulating different bile acid receptors and transporters as well as the immune response. A recent study showed that BBR-induced activation of intestinal FXR is critical to its beneficial effect on hepatic metabolism [63]. The inhibition of the WDSW-induced increase in body weight is likely linked to its effect on gut microbiome since it has no effect on food intake. Our ongoing project is examining the mechanisms via which BBR modulates gut microbiome and bile acid metabolism.

**Figure 8:**
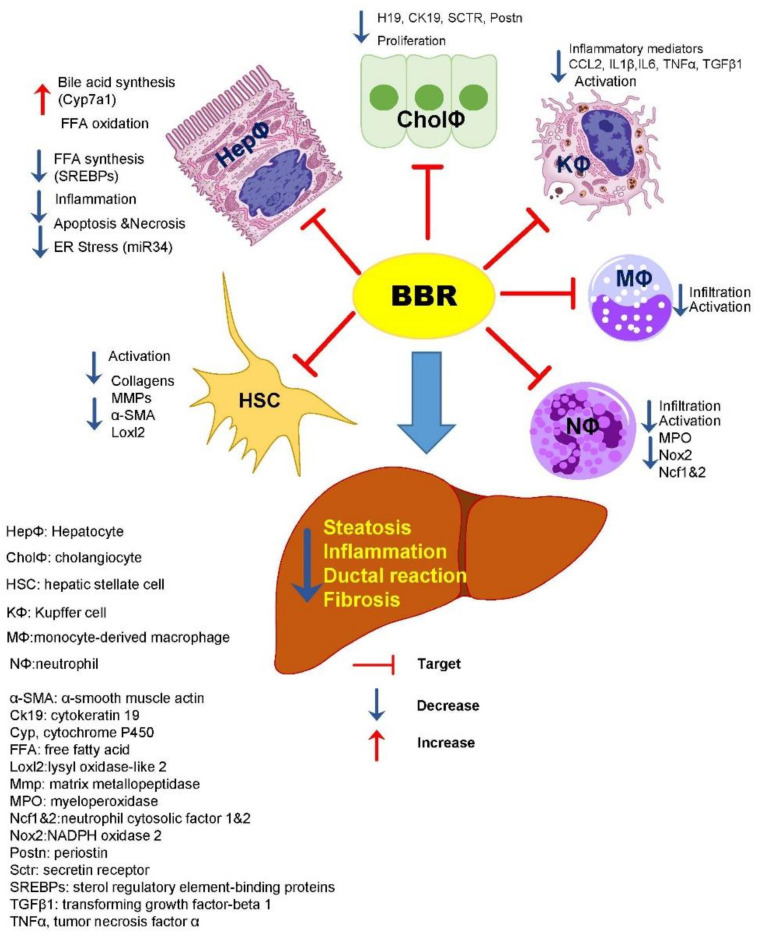
Schematic depiction of major targets of BBR in preventing NASH disease progression. During the NASH disease progression, lipid is accumulated due to the disruption of hepatic metabolic homeostasis induction of stress response in hepatocytes. Hepatocyte injury-induced immune cell infiltration and activation (monocytes, macrophages, neutrophils) further promotes the activation of hepatic stellate cells (HSC) and the proliferation of cholangiocytes. BBR can reduce metabolic stress in hepatocytes and inhibition of inflammation by reducing macrophage and neutrophil infiltration and activation. It also inhibits HSC and cholangiocyte activation. Overall, BBR effectively prevents NASH progression from NAFL by modulating multiple pathways.

In summary, this study provided comprehensive information for understanding the cellular and molecular mechanisms of BBR as a potential preventive and therapeutic agent for NASH. As illustrated in Figure 8, BBR can directly or indirectly target hepatocytes, macrophages, neutrophils, stellate cells, and cholangiocytes and modulate multiple pathways related to lipid and bile acid metabolism, inflammation, oxidative stress response, innate immune response, and fibrotic response. These studies strongly indicate that BBR is worthy of future clinical studies.

## Data Availability

The data presented in this study are available on request from the corresponding author. The data are not publicly available due to incomplete analysis of all the data.

## Supplementary Materials

The following are available online at https://www.mdpi.com/2073-4409/10/2/210/s1, Figure S1: Effect of BBR on food intake, serum biochemical parameters, and bile acid profiles in the WDSW-induced NAFLD mouse model, Figure S2: Effect of BBR on NASH progression in the WDSW-induced NAFLD mouse model, Figure S3: Venn diagram of DEGs of the two comparisons: WDSW vs. ND and WDSW + BBR vs. WDSW, Figure S4: Fatty acid elongation pathway of the two comparisons: WDSW vs. ND and WDSW + BBR vs. WDSW, Figure S5: Heatmap of genes involved in inflammation and stress associated with NASH and mRNA levels of stress-related genes, Figure S6: Effect of BBR on neutrophil activation associated with NASH, Figure S7: Oxidative phosphorylation pathway of the two comparisons: WDSW vs. ND and WDSW + BBR vs. WDSW, Figure S8: Primary bile acid biosynthesis pathway of the two comparisons: WDSW vs. ND and WDSW + BBR vs. WDSW, Figure S9: Bile secretion pathway of the two comparisons: WDSW vs. ND and WDSW + BBR vs. WDSW, Figure S10: Heatmap of genes involved in bile acid metabolism associated with NASH, Figure S11: Effect of BBR on hepatic bile acid profiles in the WDSW-induced NAFLD mouse model, Figure S12: Heatmap of genes involved in hepatic fibrosis associated with NASH and mRNA expression levels of genes involved in cholangiocyte proliferation. Table S1: Bile acids contents in the serum (Mean ± SD, µmol/L), Table S2: Bile acid profile in the serum (Mean ± SD, µmol/L), Table S3: Bile acids contents in the liver (Mean ± SD, pmol/mg liver), Table S4: Bile acid profile in the liver (Mean ± SD, pmol/mg liver), Table S5: Western Diet (TD88137), Table S6: List of antibodies, Table S7: List of bile acid standards, Table S8: LC-MS/MS parameters for the bile acids analyzed in this study.

## Abbreviations

NAFLD, nonalcoholic fatty liver disease; NAFL, simple steatosis; NASH, nonalcoholic steatohepatitis; BBR, berberine; ER, endoplasmic reticulum; HSC, hepatic stellate cells; HuR, human antigen R; HFD, high fat diet; HCC, hepatocellular carcinoma; WD, Western diet; SW, fructose/glucose solution; ND, normal diet; NW, normal water; WDSW, Western diet plus fructose/glucose solution; RNAseq, RNA sequencing; FC, fold change; GO, Gene Ontology; BP, biological process; CC, cellular component; MF, molecular function; KEGG, Kyoto Encyclopedia of Genes and Genomes; AST, aspartate aminotransferase; ALT, alanine aminotransferase; ALP, alkaline phosphatase; TC, total cholesterol; TG, triglycerol; VLDL, very-low-density lipoprotein; TCA, taurocholic acid; BA, bile acids; H&E, hematoxylin and eosin; RT-PCR, reverse-transcription polymerase chain reaction; Fasn, fatty acid synthase; Acc1, acetyl CoA carboxylase; Elovl7, elongation of very-long-chain fatty acids member 7; Fads2, fatty acid desaturase 2; Scd1, stearoyl-coenzyme A desaturase 1; Ces2α, carboxylesterase 2α; Lpl, lipoprotein lipase; Nceh1, neutral cholesterol ester hydrolase 1; Pnpla3, patatin-like phospholipase domain containing 3; Srebp1 and 2, sterol regulatory element-binding protein-1 and 2; IHC, immunohistochemistry; MPO, myeloperoxidase; IL-6, interleukin 6; Tnfα, tumor necrosis factor α; TMEM173/STING, transmembrane protein 173; Nox2, NADPH oxidase 2; Ntcp, sodium/bile acid cotransporter; Bsep, bile salt export protein; Cyp, cytochrome P450; Sirt1, sirtuin 1; Mmp, matrix metallopeptidase; Tgfβ1, transforming growth factor-β1; Ck19, cytokeratin 19; Postn, periostin; Sctr, secretin receptor; Sox4, SRY (sex determining region Y)-box 4; Sphk2, sphingosine kinase 2; DCA, deoxycholic acid; CA, cholic acid.
