# Supplementary material for: Berberine Prevents Disease Progression of Nonalcoholic Steatohepatitis through Modulating Multiple Pathways
Source: Cells. 2021 Jan 21;10(2):210. doi: 10.3390/cells10020210 (PMC7912096; doi:10.3390/cells10020210)
Supplement: Supplementary file 1 [file cells-10-00210-s001.pdf]

Supplementary Materials

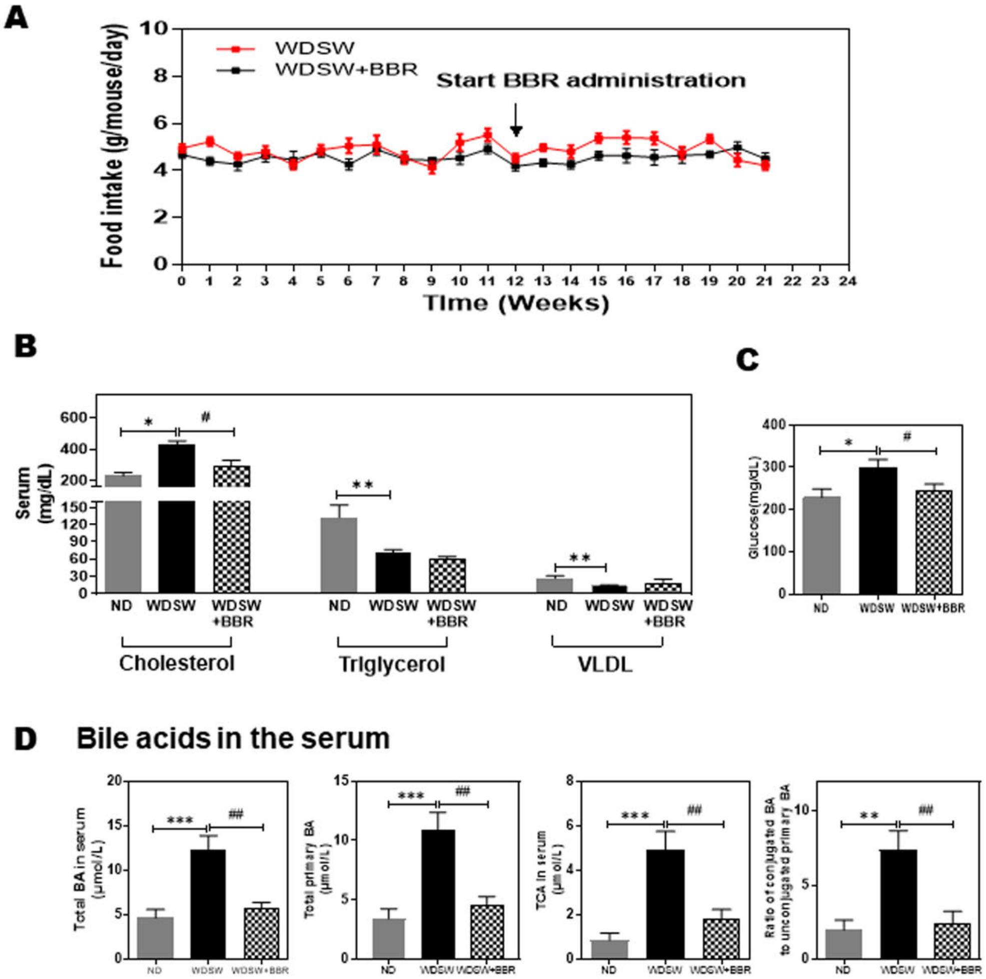

Figure S1. Effect of BBR on food intake, serum biochemical parameters, and bile acid profiles in Table 6. mice were fed a normal chow diet with tap water (ND) or Western Diet with high fructose/glucose (WDSW) for 12 weeks. WDSW animals were treated with vehicle ($n = 10$) or BBR (50 mg/kg/day, $n = 11$) via oral gavage once daily for 9 weeks, while continuing feeding with WDSW. ND mice ($n = 9$) did not receive any treatment. (**A**) The average food intake amount of WDSW and WDSW + BBR mice during the experimental feeding period of 21 weeks. Data are expressed as the mean ± SEM. (**B**) Serum lipid analysis (total cholesterol, triglyceride, very-low-density lipoprotein (VLDL)). (**C**) Serum level of glucose. (**D**) Total bile acids, total primary bile acids, TCA, and ratio of conjugated to unconjugated primary bile acids in the serum. Data are expressed as the mean ± SEM. Statistical significance: * $p < 0.05$ vs. ND, ** $p < 0.01$ vs. ND, *** $p < 0.001$ vs. ND; # $p < 0.05$ vs. WDSW, ## $p < 0.01$ vs. WDSW; ns = nonsignificant. Abbreviation: BA, bile acids.

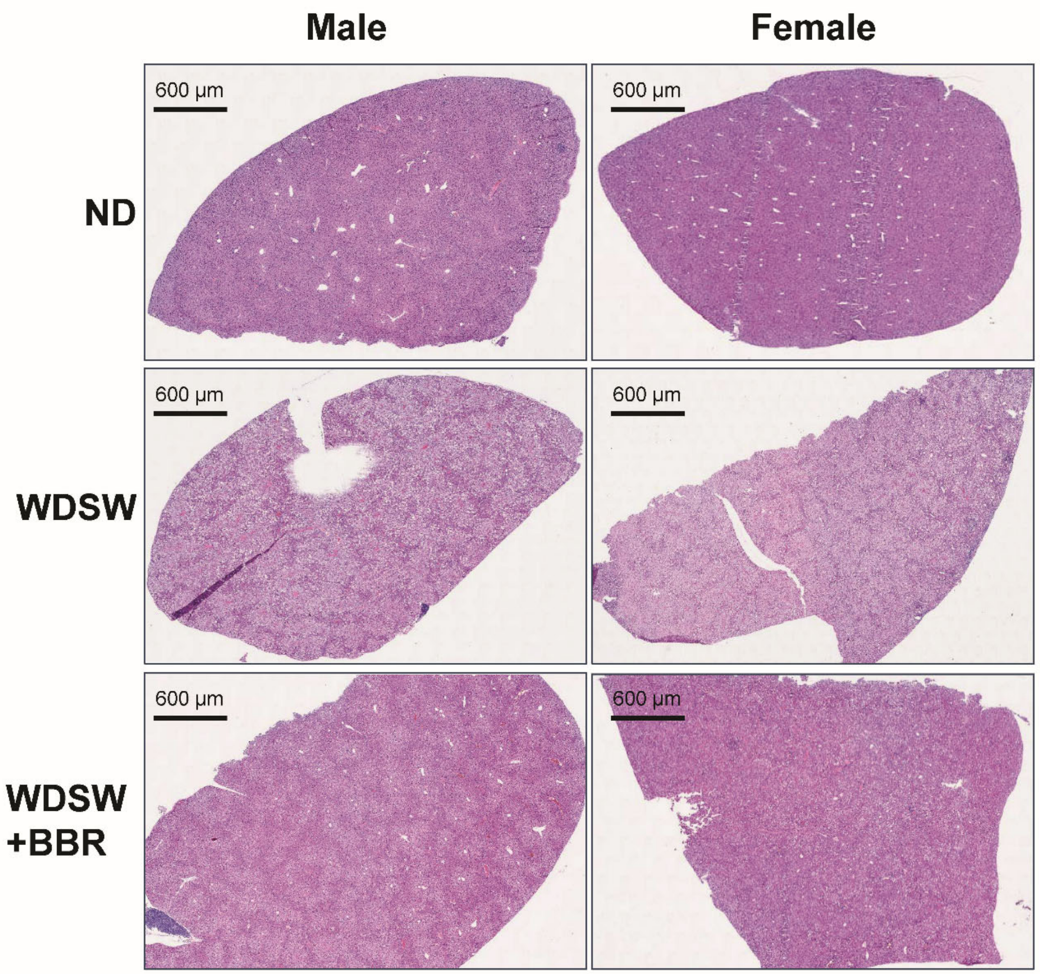

Figure 2. Effect of BBR on NASH progression in the WDSW-induced NAFLD mouse model. Representative images of whole liver sections stained with H&E (scale bar, 600 μm, 1.8× magnification) in both male and female mice using a Vectra Polaris Automated Quantitative Pathology Imaging System. Abbreviation: H&E, hematoxylin and eosin.

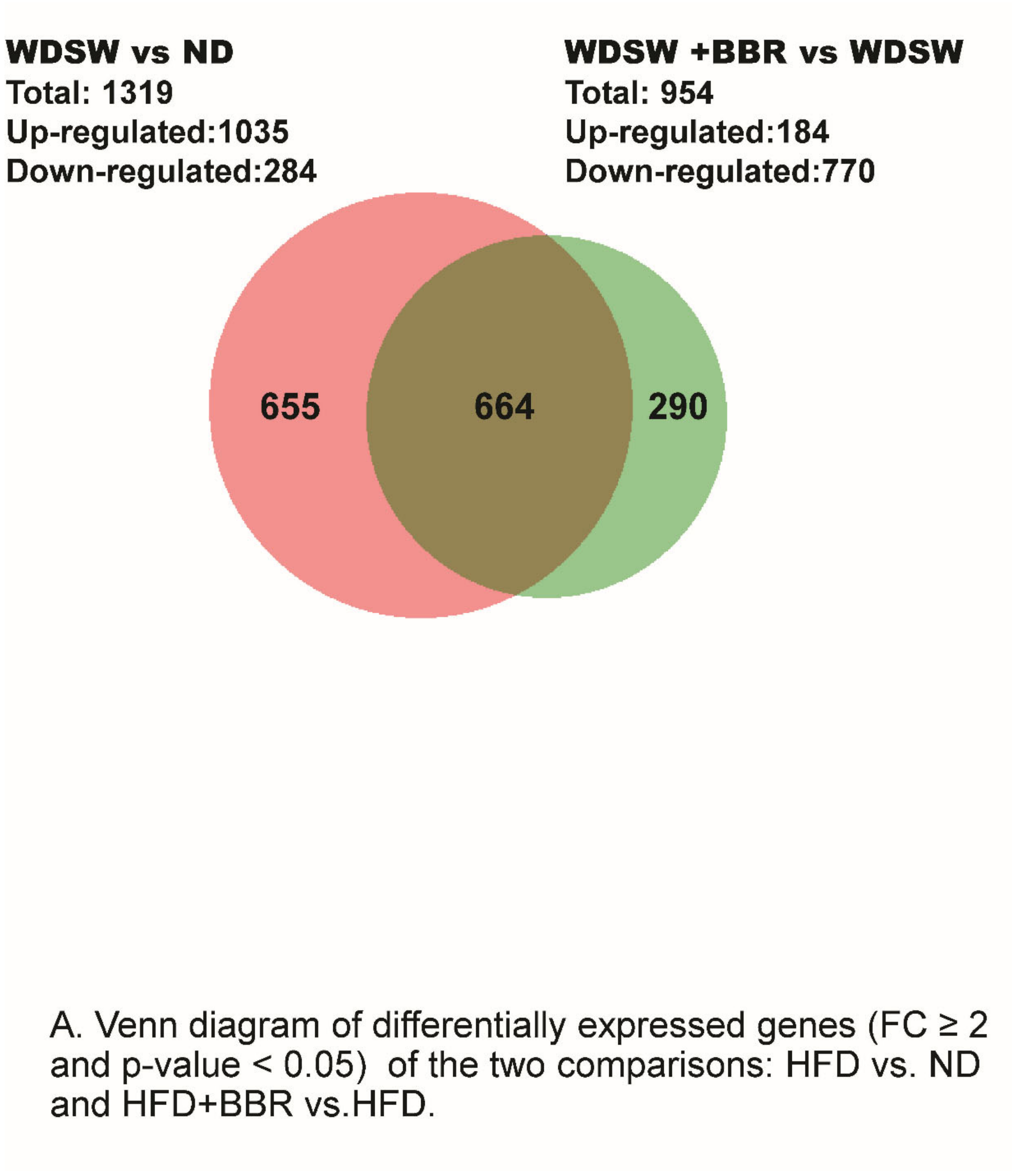

Figure S3. Venn diagram of DEGs of the two comparisons: WDSW vs. ND and WDSW + BBR vs. WDSW. Venn diagram of DEGs of the two comparisons: WDSW vs. ND and WDSW + BBR vs. WDSW. WDSW vs. ND: total of 1319 DEGs including 1035 upregulated genes and 284 downregulated genes; WDSW + BBR vs. WDSW: total of 954 DEGs including 184 upregulated genes and 770 downregulated genes. There were a total of 664 DEGs in the two comparisons.

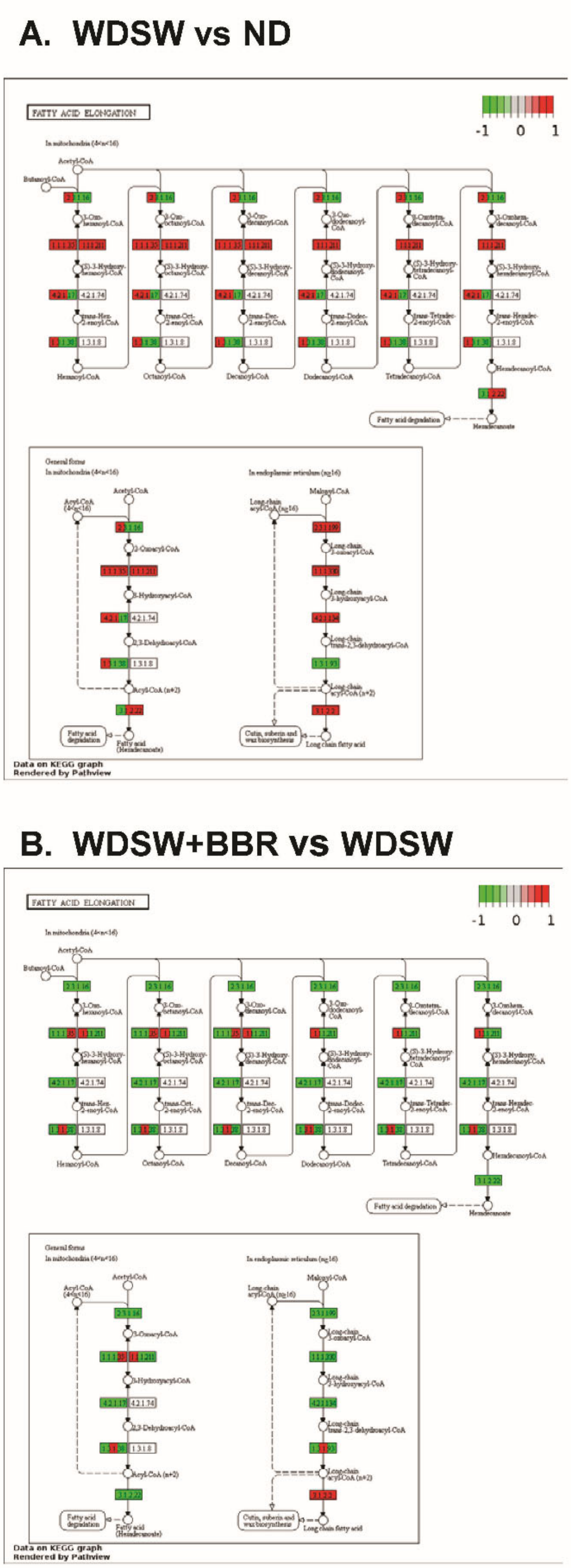

Figure S4. Fatty acid elongation pathway of the two comparisons: WDSW vs. ND and WDSW + BBR vs. WDSW. RNAseq data were used to functionally analyze and map genes involved in the fatty acid elongation pathway using Kyoto Encyclopedia of Genes and Genomes (KEGG). (**A**)

Fatty acid elongation pathway in WDSW vs. ND. (**B**) Fatty acid elongation pathway in WDSW + BBR vs. WDSW. Red and green colors indicate upregulated and downregulated gene expression, respectively.

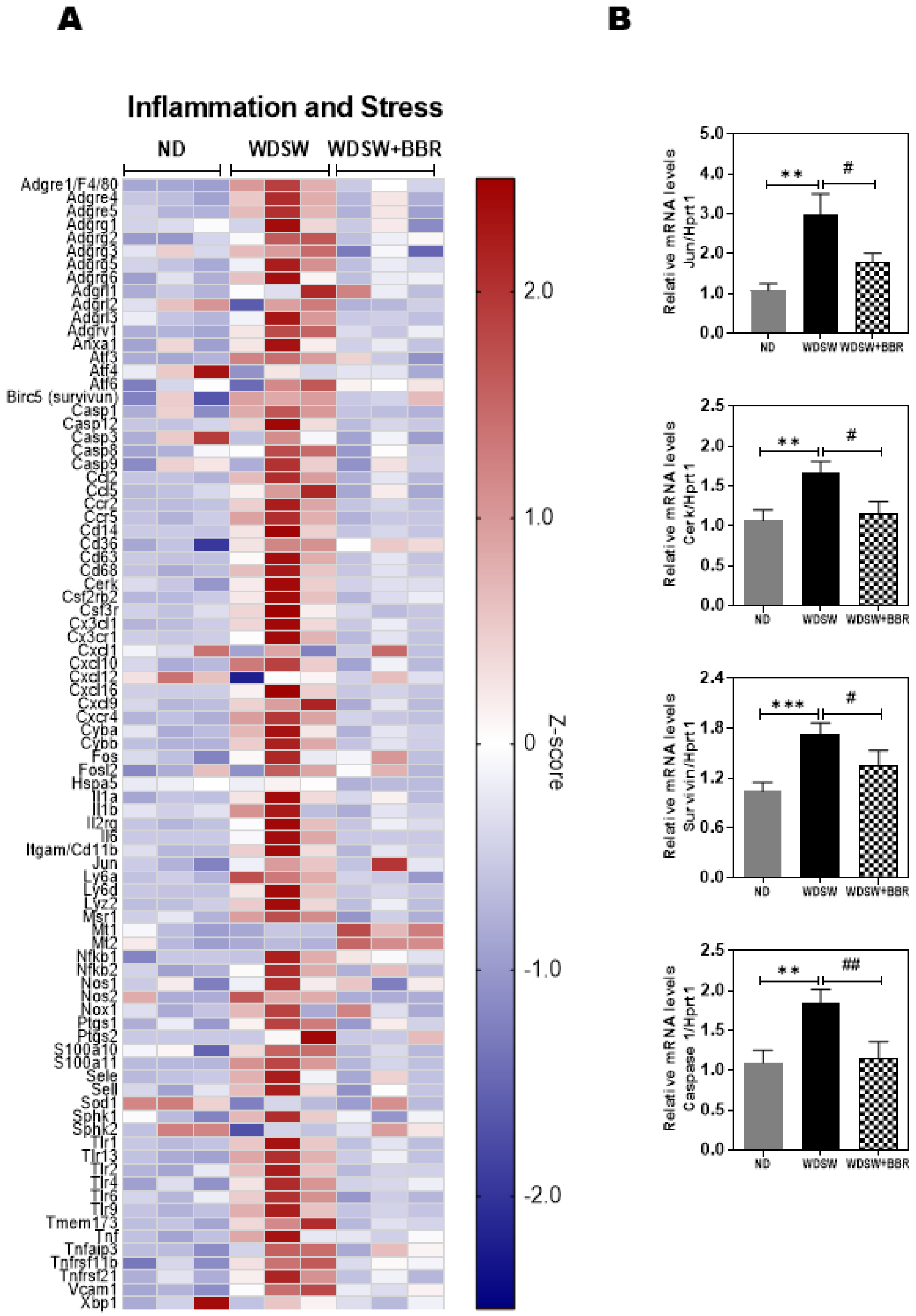

Figure S5. Heatmap of genes involved in inflammation and stress associated with NASH and mRNA levels of stress-related genes. **A**. Heatmap of genes involved in inflammation and stress associated with NASH. A *Z*-score was calculated for the RNAseq data to normalize tag counts. Red and blue colors indicate high and low gene expression, respectively. **B**.

Relative mRNA levels of genes involved in stress response: Jun, Cerk, Survivin, and Caspase 1. Data are expressed as the mean ± SEM. Statistical significance: ** $p < 0.01$ vs. ND, *** $p < 0.001$ vs. ND; # $p < 0.05$ vs. WDSW.

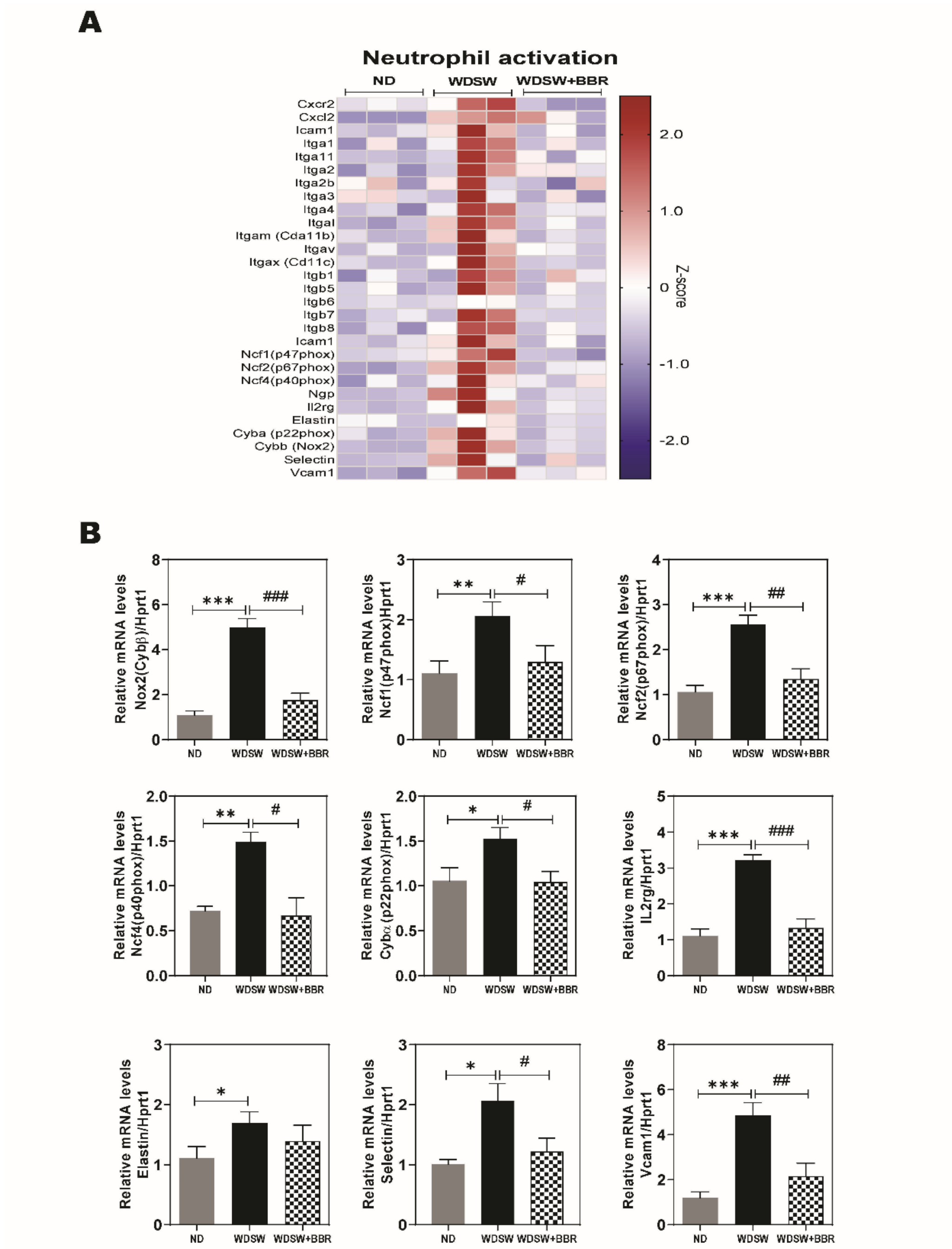

Figure S6. Effect of BBR on neutrophil activation associated with NASH. (**A**) Heatmap of genes involved in neutrophil activation associated with NASH. A Z-score was calculated for the RNAseq data to normalize tag counts. Red and blue colors indicate high and low gene expression, respectively. (**B**) Relative mRNA levels of Nox2, Ncf1, Ncf2, Ncf4, Cybα, Il2rg, Elastin, Selectin, and Vcam1 were determined by real-time RT-PCR and normalized with HPRT1 as an internal control. Data are expressed as the mean ± SEM. Statistical significance: * $p < 0.05$ vs. ND, ** $p < 0.01$ vs. ND, *** $p < 0.001$ vs. ND; $^{\#}$ $p < 0.05$ vs. WDSW, $^{\#\#}$ $p < 0.01$ vs. WDSW; $^{\#\#\#}$ $p < 0.001$ vs. WDSW.

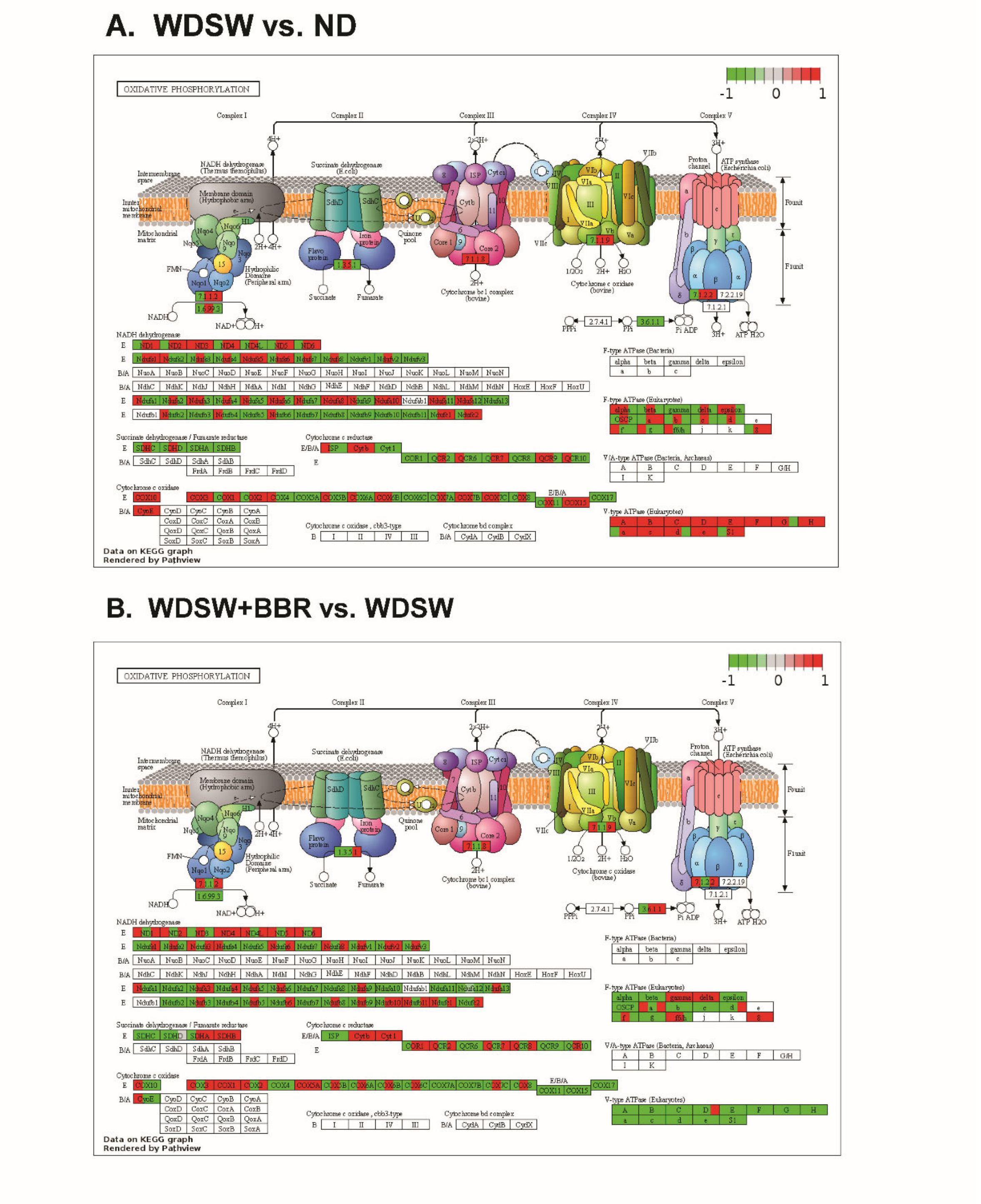

Figure S7. Oxidative phosphorylation pathway of the two comparisons: WDSW vs. ND and WDSW + BBR vs. WDSW. RNAseq data were used to functionally analyze and map genes involved in the oxidative phosphorylation pathway using Kyoto Encyclopedia of Genes and Genomes (KEGG). (**A**) Oxidative phosphorylation pathway in WDSW vs. ND. (**B**) Oxidative phosphorylation pathway in WDSW + BBR vs. WDSW. Red and green colors indicate upregulated and downregulated gene expression, respectively.

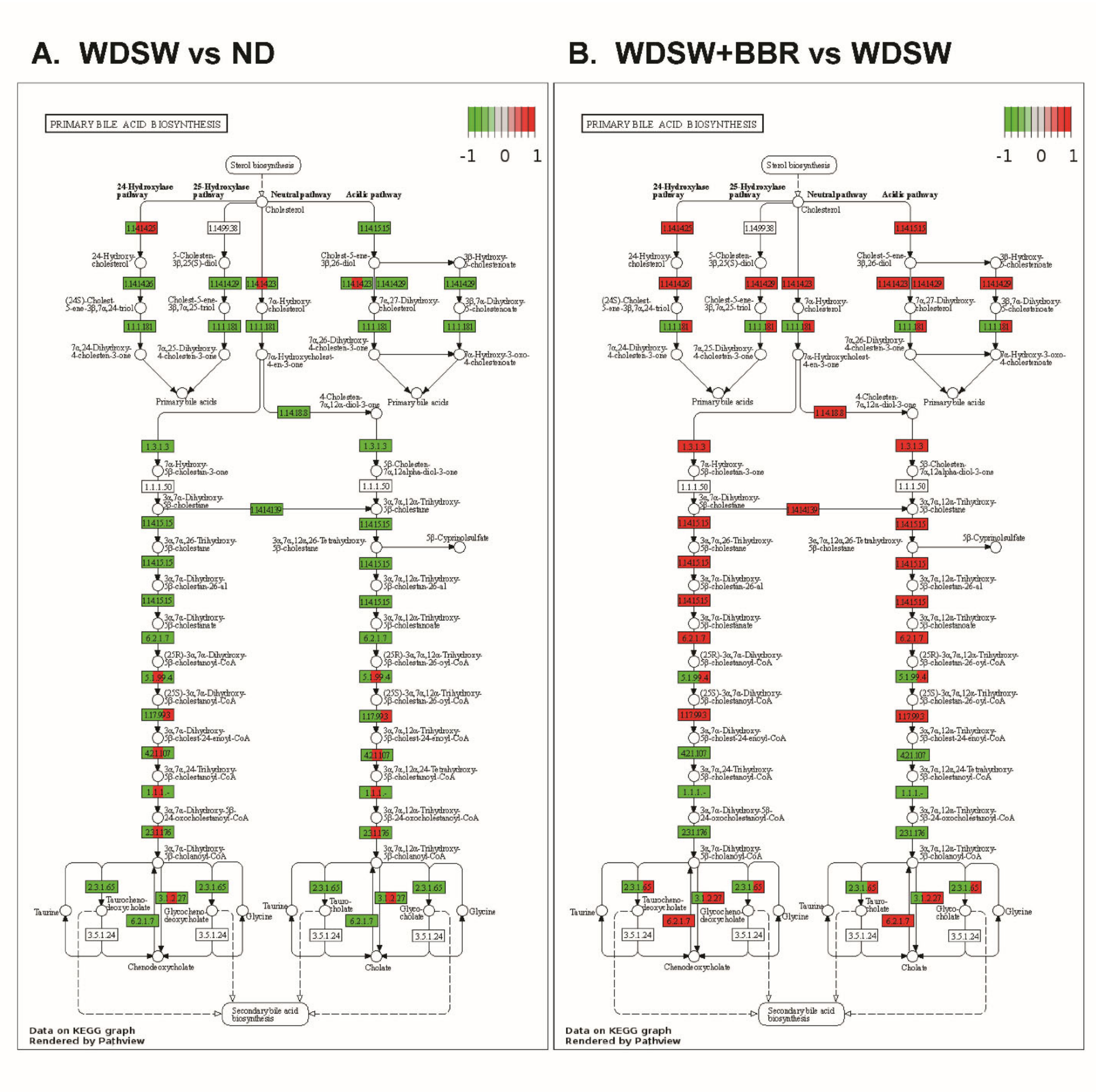

Figure S8. Primary bile acid biosynthesis pathway of the two comparisons: WDSW vs. ND and WDSW + BBR vs. WDSW. RNAseq data were used to functionally analyze and map genes involved in the primary bile acid biosynthesis pathway using Kyoto Encyclopedia of Genes and Genomes (KEGG). (**A**) Primary bile acid biosynthesis pathway in WDSW vs. ND. (**B**) Primary bile acid biosynthesis pathway in WDSW + BBR vs. WDSW. Red and green colors indicate upregulated and downregulated gene expression, respectively.

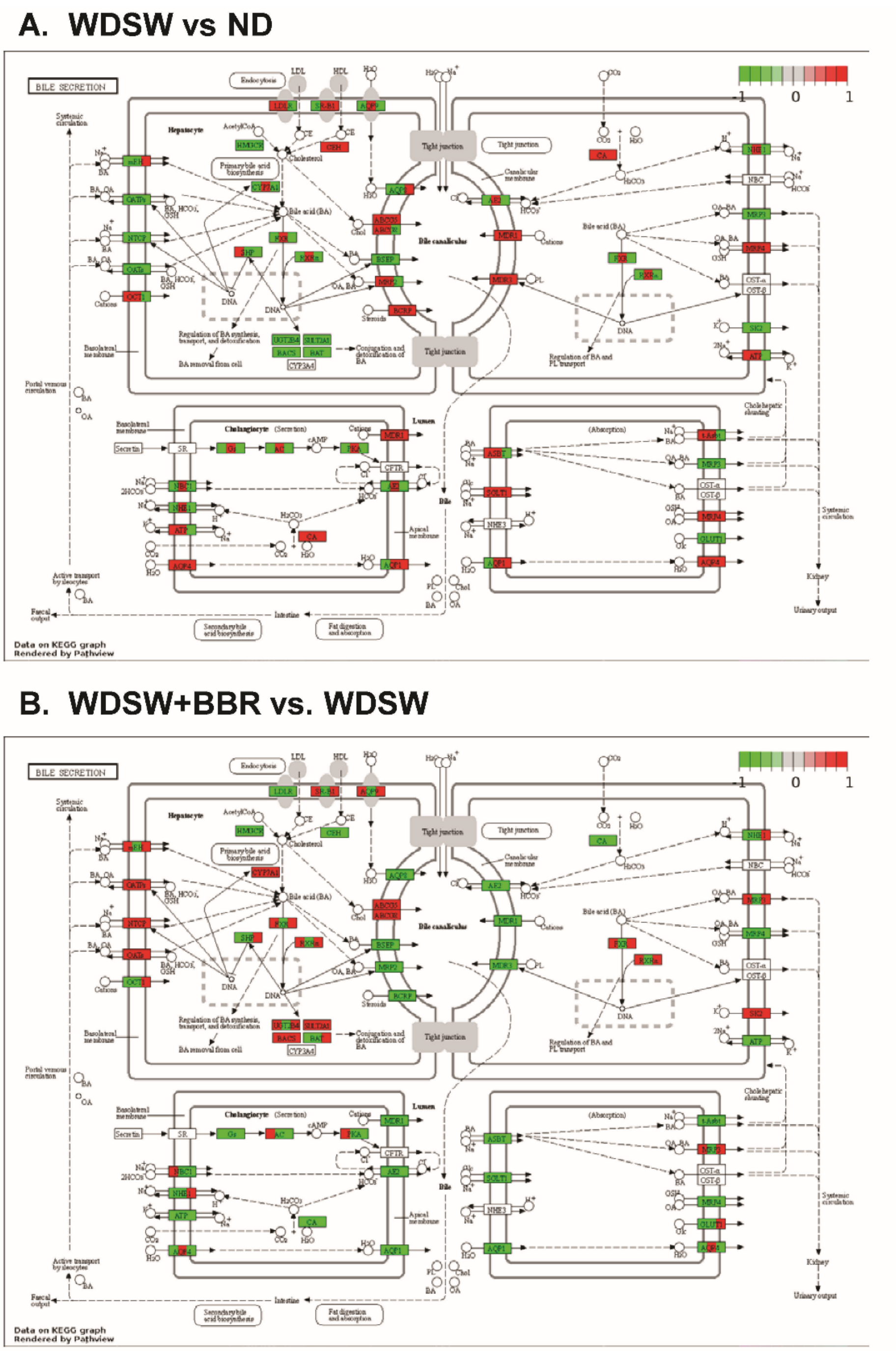

Figure S9. Bile secretion pathway of the two comparisons: WDSW vs. ND and WDSW + BBR vs. WDSW. RNAseq data were performed to functionally analyze and map genes involved in bile Scheme 10. Heatmap of genes involved in bile acid metabolism associated with NASH**.** A *Z*-score was calculated for the RNAseq data to normalize tag counts. Red and blue colors indicate high and low gene expression, respectively.

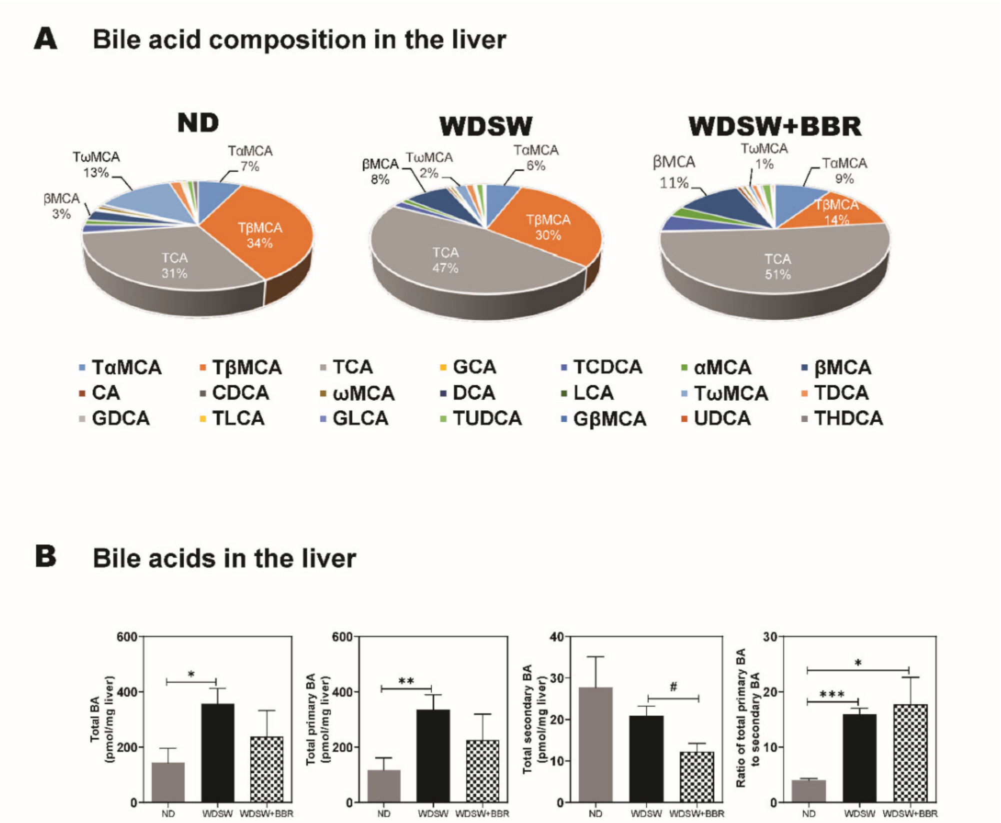

Figure S11. Effect of BBR on hepatic bile acid profiles in the WDSW-induced NAFLD mouse model. (**A**) Bile acids composition profile in the liver expressed by % of total bile acids. (**B**) Total bile acids, total primary bile acids, total Scheme 0. vs. ND, ** $p < 0.01$ vs. ND, *** $p < 0.001$ vs. ND; # $p < 0.05$ vs. WDSW. Abbreviation: BA, bile acids.

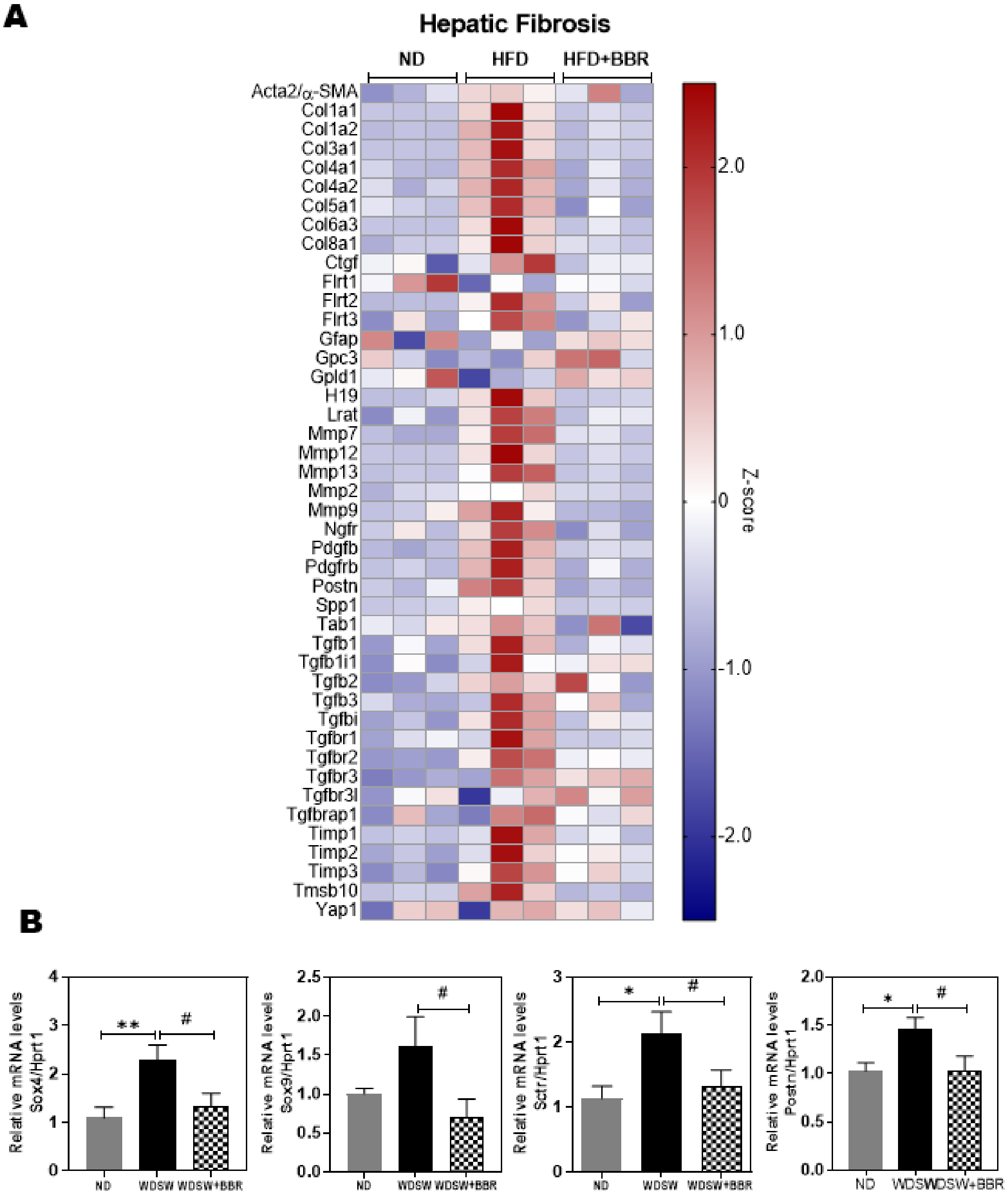

Figure S12. Heatmap of genes involved in hepatic fibrosis associated with NASH and mRNA expression levels of genes involved in cholangiocyte proliferation. (A). A *Z*-score was calculated for the RNAseq data to normalize tag counts. Red and blue colors indicate high and low gene expression, respectively. (B). Relative mRNA levels of Sox4, Sox9, Sctr, and Postn were normalized with HPRT1 as an internal control. Data are expressed as the mean ± SEM. Statistical significance: * $p < 0.05$ vs. ND, ** $p < 0.01$ vs. ND; # $p < 0.05$ vs. WDSW.

Table 1. Bile acids contents in the serum (Mean ± SD, μmol/L).

Bile Acids	ND	WDSW	WDSW + BBR
TωMCA	0.15 ± 0.11	0.26 ± 0.14	0.05 ± 0.02##
TαMCA	0.16 ± 0.03	0.75 ± 0.36*	0.35 ± 0.15#
TβMCA	0.23 ± 0.10	3.29 ± 1.81*	0.72 ± 0.59#
TUDCA	0.05 ± 0.00	0.13 ± 0.05**	0.09 ± 0.04
THDCA	0.05 ± 0.00	0.06 ± 0.01	0.04 ± 0.00#
TCA	0.20 ± 0.04	5.96 ± 3.32*	1.29 ± 0.77#
ωMCA	0.28 ± 0.15	0.18 ± 0.04	0.13 ± 0.03
αMCA	0.25 ± 0.01	0.28 ± 0.02	0.28 ± 0.04
7keto_DCA	0.10 ± 0.01	0.09 ± 0.00*	0.09 ± 0.00
βMCA	0.49 ± 0.27	0.80 ± 0.30	0.57 ± 0.44
GCA	0.06 ± 0.00	0.07 ± 0.01*	0.06 ± 0.00#
TCDCA	0.07 ± 0.01	0.23 ± 0.09*	0.13 ± 0.05#
UDCA	0.06 ± 0.01	0.06 ± 0.01	0.08 ± 0.01
TDCA	0.02 ± 0.00	0.21 ± 0.13*	0.08 ± 0.02
CA	0.08 ± 0.03	0.35 ± 0.16*	0.10 ± 0.06#
GDCA	0.07 ± 0.01	0.07 ± 0.00	0.07 ± 0.00
TLCA	0.06 ± 0.00	0.06 ± 0.00	0.06 ± 0.00
CDCA	0.07 ± 0.01	0.07 ± 0.01	0.06 ± 0.00
GLCA	0.06 ± 0.01	0.09 ± 0.03	0.09 ± 0.02
DCA	0.11 ± 0.01	0.23 ± 0.09*	0.23 ± 0.27
LCA	0.05 ± 0.00	0.05 ± 0.00	0.05 ± 0.01

Statistical significance: *$p < 0.05$ vs ND, ** $p < 0.01$ vs ND; # $p < 0.05$ vs WDSW, ## $p < 0.01$ vs DSW; Some bile acids were not detectable, including GbMCA, MDCA, HCA, HDCA, isoDCA, 12keto_LCA, isoLCA, allo_isoLCA.

TableS2. Bile acid profile in the serum (Mean ± SD, μmol/L).

**Groups.**	**ND**	**WDSW**	**WDSW + BBR**
Total BA	4.65 ± 3.00	12.33 ± 5.17***	5.67 ± 2.16##
Total primary BA	3.41 ± 2.61	10.90 ± 4.94***	4.58 ± 2.12##
Total primary conjugated BA	2.23 ± 2.40	9.50 ± 4.88***	3.27 ± 2.35##
Total primary unconjugated BA	1.19 ± 0.58	1.40 ± 0.42	1.31 ± 0.76
Total secondary BA	1.24 ± 0.42	1.43 ± 0.30	1.09 ± 0.50
Total secondary conjugated BA	0.67 ± 0.19	0.89 ± 0.25**	0.52 ± 0.18##
Total secondary unconjugated BA	0.57 ± 0.16	0.54 ± 0.12	0.57 ± 0.42
Total conjugated BA	2.89 ± 2.70	10.38 ± 5.10***	3.10 ± 1.27##
Total unconjugated BA	1.75 ± 0.72	1.94 ± 0.53	2.06 ± 1.12
Ratio of total primary BA to total BA	0.69 ± 0.09	0.87 ± 0.04***	0.79 ± 0.08#
Ratio of total primary BA to total secondary BA	2.48 ± 1.10	7.38 ± 2.41***	3.82 ± 1.76##
Ratio of total primary conjugated BA to total primary unconjugated BA	1.96 ± 2.15	7.35 ± 4.34**	2.37 ± 2.33##
Ratio of total conjugated BA to total unconjugated BA	1.68 ± 1.51	5.69 ± 3.14**	2.23 ± 2.33#
Ratio of total secondary BA to total BA	0.31 ± 0.09	0.13 ± 0.04***	0.21 ± 0.08#
Ratio of total secondary conjugated BA to total secondary unconjugated BA	1.19 ± 0.44	1.54 ± 0.56*	1.45 ± 1.42

Statistical significance: *$p< 0.05$ vs. ND, **$p< 0.01$ vs. ND, ***$p< 0.001$ vs. ND; #$p < 0.05$ vs WDSW, ##$p < 0.01$ vs WDSW.

Table S3. Bile acids contents in the liver (Mean ± SD, pmol/mg liver).

Bile Acids	ND	WDSW	**WDSW + BBR**
TαMCA	10.67 ± 9.27	21.12 ± 10.01	22.48 ± 20.03
TβMCA	50.02 ± 45.92	106.62 ± 45.38	32.46 ± 27.65#
TCA	44.96 ± 31.31	167.03 ± 54.05**	121.81 ± 100.46
GCA	0.50 ± 0.07	0.67 ± 0.09*	0.62 ± 0.21
TCDCA	3.36 ± 1.34	6.35 ± 1.96*	11.60 ± 4.39#
αMCA	2.05 ± 0.44	4.06 ± 1.46*	7.32 ± 6.07
βMCA	4.61 ± 1.92	26.89 ± 14.16*	26.68 ± 27.60
CA	0.48 ± 0.07	1.57 ± 0.47**	1.56 ± 1.35
CDCA	0.44 ± 0.06	0.57 ± 0.05**	0.77 ± 0.18
ωMCA	1.35 ± 0.33	1.65 ± 0.35	1.29 ± 0.42
DCA	0.80 ± 0.66	0.55 ± 0.07	0.54 ± 0.11
LCA	0.35 ± 0.06	0.37 ± 0.01	0.39 ± 0.05
TωMCA	18.99 ± 12.57	6.93 ± 2.06*	1.48 ± 0.81#
TDCA	2.73 ± 0.61	4.42 ± 1.63	2.03 ± 3.18
GDCA	0.50 ± 0.07	0.54 ± 0.03	0.55 ± 0.08
TLCA	0.54 ± 0.07	0.59 ± 0.04	0.59 ± 0.09
GLCA	0.48 ± 0.09	0.74 ± 0.23	0.85 ± 0.22
TUDCA	1.39 ± 0.57	3.83 ± 1.68*	3.42 ± 2.36
GβMCA	nd	0.59 ± 0.04	0.62 ± 0.12
UDCA	nd	0.49 ± 0.11	0.72 ± 0.39

Statistical significance: * $p < 0.05$ vs ND, ** $p < 0.01$ vs ND; # $p < 0.05$ vs WDSW. Some bile acids were not detectable, including GbMCA, MDCA, HCA, HDCA, isoDCA, 12keto_LCA, isoLCA, allo_isoLCA. nd, not detectable.

Table S4. Bile acid profile in the liver(Mean ± SD, pmol/mg liver).

**Group**	**ND**	**WDSW**	**WDSW + BBR**
Total BA	144.85 ± 102.94	356.33 ± 126.15*	237.82 ± 190.05
Total primary BA	117.08 ± 88.55	335.47 ± 121.26**	225.60 ± 187.51
Total primary conjugated BA	109.50 ± 86.44	302.38 ± 108.79*	189.27 ± 152.36
Total primary unconjugated BA	7.58 ± 2.44	33.09 ± 15.81*	36.33 ± 35.17
Total secondary BA	27.77 ± 14.70	20.86 ± 5.36	12.22 ± 4.04#
Total secondary conjugated BA	25.26 ± 14.42	17.88 ± 5.02	9.29 ± 3.41#
Total secondary unconjugated BA	2.50 ± 0.79	2.97 ± 0.57	2.93 ± 0.87
Total conjugated BA	134.76 ± 100.62	320.27 ± 113.38*	198.56 ± 154.06
Total unconjugated BA	10.08 ± 2.72	36.06 ± 16.30*	39.26 ± 36.01
Ratio of total primary BA to total BA	0.79 ± 0.03	0.94 ± 0.01	0.93 ± 0.04
Ratio of total primary BA to total secondary BA	3.95 ± 0.84	15.90 ± 2.53***	13.22 ± 4.84
Ratio of total primary conjugated BA to total primary unconjugated BA	13.30 ± 6.57	9.93 ± 3.86	5.70 ± 0.72
Ratio of total conjugated BA to total unconjugated BA	12.38 ± 6.28	9.56 ± 3.59	5.45 ± 0.62
Ratio of total secondary BA to total BA	0.21 ± 0.03	0.06 ± 0.01***	0.07 ± 0.04
Ratio of total secondary conjugated BA to total secondary unconjugated BA	10.15 ± 5.38	6.08 ± 1.51	3.18 ± 0.95#

Statistical significance: * $p < 0.05$ vs. ND, ** $p < 0.01$ vs. ND, *** $p < 0.001$ vs. ND; # $p < 0.05$ vs WDSW.

Table S5. Western Diet (TD88137).

Formula	Kg g/Kg
Casein	195.0
DL-Methionine	3.0
Sucrose	341.46
Corn Starch	150.0
Andydrous Milkfat	210.0
Cholesterol	1.5
Cellulose	50.0
Mineral Mix, AIN-76 (170915)	35.0
Calcium Carbonate	4.0
Vitamin Mix, Teklad (40060)	10.0
Ethoxyquin	0.04

TableS 6. List of antibodies.

Antibody	Species	Source	Catalog #	Application/ dilution
CK19 (TROMA-III)	Rat	DSHB University of lowa	TROMA-III	IHC (1:50)
CYP7A1	Mouse	Santa Cruz	sc-518007	WB (1:500)
F4/80	Rabbit	Cell Signaling	70076S	IHC (1:200)
Histone H3	Rabbit	Cell Signaling	9715S	WB (1:1000)
Lamin B	Mouse	Santa Cruz	sc-374015	WB (1:500)
MPO	Rabbit	Biocare Medical	SKU:023	IHC (1:1)
SREBP-1	Mouse	Santa Cruz	sc-365513	WB (1:500)
SREBP-2	Rabbit	Abcam	ab30682	WB (1:200)
β-actin (JLA20)	Mouse	DSHB University of lowa	JLA20	WB (1:500)
anti-Rabbit IgG (H + L)-HRP	Goat	Invitrogen	365-6120	WB(1:2500)
anti-Mouse IgG(H + L)-HRP	Goat	Bio-RAD	170-6516	WB(1:2500)
anti-RatIgG Antibody (H + L),Biotinylated	Rabbit	Vector Laboratories	BA-4000	IHC (1:1000)

Table S7. List of bile acid standards.

Abbreviation	Full name	Vendor
CA	Cholic acid	Sigma-Aldrich, Inc.
d_4-CA	[2,2,4,4]-d_4-cholic acid	Steraloids, Inc.
GCA	Glycocholic acid	Sigma-Aldrich, Inc.
d_4-GCA	[2,2,4,4]-d_4-Glycocholic acid	Cayman Chemical Co.
TCA	Taurocholic acid	Sigma-Aldrich, Inc.
d_4-TCA	[2,2,4,4-d_4]--Taurocholic acid	Cayman Chemical Co.
CDCA	Chenodeoxycholic acid	Sigma-Aldrich, Inc.
d_4-CDCA	d_4-Chenodeoxycholic acid	Steraloids, Inc.
GCDCA	Glycochenodeoxycolic acid	Sigma-Aldrich, Inc.
TCDCA	Taurochenodeoxycholic acid	Sigma-Aldrich, Inc.
d_4-TCDCA	d_4-Taurochenodeoxycholic acid	Sigma-Aldrich, Inc.
UDCA	Ursodeoxycholic acid	Sigma-Aldrich, Inc.
GUDCA	Glycourosodeoxycholic acid	Sigma-Aldrich, Inc.
TUDCA	Tauroursodeoxycholic acid	Sigma-Aldrich, Inc.
DCA	Deoxycholic acid	Sigma-Aldrich, Inc.
d_4-DCA	d_4-Deoxycholic acid	Cayman Chemical Co.
isoDCA	Isodeocycholic acid	Steraloids Inc.
GDCA	Glycodeoxycholic acid	Sigma-Aldrich, Inc.
d_4-GDCA	d_4-Glycodeoxycholic acid	Sigma-Aldrich, Inc.
TDCA	Taurodeoxycholic acid	Sigma-Aldrich, Inc.

LCA	Lithocholic acid	Sigma-Aldrich, Inc.
d_4-LCA	d_4-Lithocholic acid	Cayman Chemical Co.
GLCA	Glycolothocholic acid	Steraloids Inc.
d_4-GLCA	Glycolothocholic acid	Sigma-Aldrich, Inc.
TLCA	Taurolithocholic acid	Steraloids Inc.
d_4-TLCA	d_4-Taurolithocholic acid	Sigma-Aldrich, Inc.
isoLCA	Isolithocholic acid	Steraloids Inc.
iso-alloLCA	Isoallolithocholic acid	Steraloids Inc.
HCA	Hyocholic acid	Steraloids Inc.
GHCA	Glycohyodeoxycholic acid	Cayman Chemical Co.
THCA	Taurohyocholic acid	Cayman Chemical Co.
αMCA	α-Muricholic acid	Steraloids Inc.
T-αMCA	Tauro α-muricholic acid	Steraloids Inc.
βMCA	β-Muricholic acid	Steraloids Inc.
G-βMCA	Glyco β-muricholic acid	Cayman Chemical Co.
T-βMCA	Tauro β-muricholic acid	Cayman Chemical Co.
ωMCA	ω-Muricholic acid	Cayman Chemical Co.
T-ωMCA	Tauro ω-muricholic acid	Cayman Chemical Co.
HDCA	Hyodeoxycholic acid	Sigma-Aldrich, Inc.
MDCA	Murideocycholic acid	Steraloids Inc.
GHDCA	Glycohyodeoxycholic acid	Cayman Chemical Co.
THDCA	Taurohyodeoxycholic acid	Steraloids Inc.
DhLCA	Dehydrolithocholic acid	Steraloids Inc.
7-KetoDCA	7-Ketodeoxycholic acid	Steraloids Inc.
7-KetoLCA	7-Ketolithocholic acid	Steraloids Inc.
12-KetoLCA	12-Ketolithocholic acid	Steraloids Inc.

Table S8. LC-MS/MS parameters for the bile acids analyzed in this study.

Abbreviation	Bile Acid Name	MRM (*m/z*)	CE (eV)	R.T. (min)
CA	Cholic acid<br>: 3α,7α,12α-Trihydroxy-5β-cholan-24-oic acid	407.3 > 343.3	34	13.71
d_4-CA	[2,2,4,4]-d_4-cholic acid<br>: 3α,7α,12α-Trihydroxy-[2,2,4,4]-d_4-5β-cholan-24-oic acid	411.3 > 343.1	34	13.71
GCA	Glycocholic acid<br>: Glyco 3α,7α,12α-trihydroxy-5β-cholan-24-oic acid	464.5 > 74.2	52	10.88
d_4-GCA	[2,2,4,4]-d_4-Glycocholic acid<br>: Glyco 3α,7α,12α-trihydroxy-[2,2,4,4-d_4]-5β-cholan-24-oic acid	468.3 > 73.95	45	10.87
TCA	Taurocholic acid<br>: Tauro 3α,7α,12α-trihydroxy-5β-cholan-24-oic acid	514.3 > 124.0	56	9.39
d_4-TCA	Taurocholic acid<br>: Tauro 3α,7α,12α-trihydroxy-[2,2,4,4-d_4]-5β-cholan-24-oic acid	518.3 > 124.1	57	9.38
CDCA	Chenodeoxycholic acid<br>: 3α,7α-Dihydroxy-5β-cholan-24-oic acid	391.3 > 391.3	20	16.76
d_4-CDCA	d_4-Chenodeoxycholic acid<br>: 3α,7α-Dihydroxy-[2,2,4,4-d_4]-5β-cholan-24-oic acid	395.3 > 395.3	20	16.75
GCDCA	Glycochenodeoxycolic acid<br>: Glyco 3α,7α-dihydroxy-5β-cholan-24-oic acid	448.3 > 74.2	34	13.77
TCDCA	Taurochenodeoxycholic acid<br>: Tauro 3α,7α-dihydroxy-5β-cholan-24-oic acid	498.3 > 80.0	76	12.39
d_4-TCDCA	d_4-Taurochenodeoxycholic acid<br>: Tauro 3α,7α-dihydroxy-[2,2,4,4-d_4]-5β-cholan-24-oic acid	502.3 > 79.9	65	12.37
UDCA	Ursodeoxycholic acid	391.3 > 345.2	35	12.68

	: 3α,7β-Dihydroxy-5β-cholan-24-oic acid			
GUDCA	Glycourosodeoxycholic acid<br>: Glyco 3α,7β-dihydroxy-5β-cholan-24-oic acid	448.3 > 74.1	45	9.49
TUDCA	Tauroursodeoxycholic acid<br>: Tauro 3α,7β-Dihydroxy-5β-cholan-24-oic acid	498.3 > 80.0	65	7.93
DCA	Deoxycholic acid<br>: 3α,12α-Dihydroxy-5β-cholan-24-oic acid	391.3 > 345.2	35	17.15
d_4-DCA	d_4-Deoxycholic acid<br>: 3α,12α-Dihydroxy-[2,2,4,4-d_4]-5β-cholan-24-oic acid	395.3 > 395.3	20	17.13
isoDCA	Isodeocycholic acid<br>: 3β,12α-Dihydroxy-5β-cholan-24-oic acid	391.3 > 391.3	20	13.98
GDCA	Glycodeoxycholic acid<br>: Glyco 3α,12α-dihydroxy-5β-cholan-24-oic acid	448.3 > 74.1	45	14.42
d_4-GDCA	d_4-Glycodeoxycholic acid<br>: Glyco 3α,12α-dihydroxy-[2,2,4,4-d_4]-5β-cholan-24-oic acid	452.3 > 74.1	53	14.40
TDCA	Taurodeoxycholic acid<br>: Tauro 3α,12α-dihydroxy-5β-cholan-24-oic acid	498.3 > 124.0	57	13.12
LCA	Lithocholic acid<br>: 3α-Hydroxy-5β-cholan-24-oic acid	375.3 > 375.3	20	19.87
d_4-LCA	d_4-Lithocholic acid<br>: 3α-Hydroxy-[2,2,4,4-d_4]-5β-cholan-24-oic acid	379.3 > 379.3	20	19.85
GLCA	Glycolothocholic acid<br>: Glyco 3α-hydroxy-5β-cholan-24-oic acid	432.3 > 74.1	50	17.03
d_4-GLCA	Glycolothocholic acid<br>: Glyco 3α-hydroxy-[2,2,4,4-d_4]-5β-cholan-24-oic acid	432.3 > 74.1	50	17.01
TLCA	Taurolithocholic acid<br>: Tauro 3α-hydroxy-5β-cholan-24-oic acid	482.3 > 80.0	65	16.08
d_4-TLCA	d_4-Taurolithocholic acid<br>: Tauro 3α-hydroxy-[2,2,4,4-d_4]-5β-cholan-24-oic acid	482.3 > 80.0	65	16.05
isoLCA	Isolithocholic acid<br>: 3β-Hydroxy-5β-cholan-24-oic acid	375.3 > 375.3	20	18.41
iso-alloLCA	Isoallolithocholic acid<br>: 3β-Hydroxy-5α-cholan-24-oic acid	375.3 > 375.3	20	18.77
HCA	Hyocholic acid<br>: 3α,6α,7α-Trihydroxy-5β-cholan-24-oic acid	407.3 > 407.3	21	12.40
GHCA	Glycohyodeoxycholic acid<br>: Glyco 3α,6α,7α-trihydroxy-5β-cholan-24-oic acid	464.3 > 74.0	45	9.06
THCA	Taurohyocholic acid<br>: Tauro 3α,6α,7α-trihydroxy-5β-cholan-24-oic acid	514.3 > 124.1	57	8.42
αMCA	α-Muricholic acid<br>: 3α,6β,7α-Trihydroxy-5β-cholan-24-oic acid	407.2 > 407.2	21	10.40
T-αMCA	Tauro α-muricholic acid<br>: Tauro 3α,6β,7α-trihydroxy-5β-cholan-24-oic acid	514.4 > 80.1	65	5.10
βMCA	β-Muricholic acid<br>: 3α,6β,7β-Trihydroxy-5β-cholan-24-oic acid	407.3 > 407.3	21	10.74
G-βMCA	Glyco β-muricholic acid<br>: Glyco 3α,6β,7β-Trihydroxy-5β-cholan-24-oic acid	464.3 > 73.95	45	7.14
T-βMCA	Tauro β-muricholic acid<br>: Tauro 3α,6β,7β-Trihydroxy-5β-cholan-24-oic acid	487.3 > 124.3	57	5.27
ωMCA	ω-Muricholic acid<br>: 3α,6α,7β-Trihydroxy-5β-cholan-24-oic acid	407.3 > 407.3	21	10.21
T-ωMCA	Tauro ω-muricholic acid	514.4 > 80.0	65	4.97

	: Tauro 3α,6α,7β-Trihydroxy-5β-cholan-24-oic acid			
HDCA	Hyodeoxycholic acid<br>: 3α,6α-Dihydroxy-5β-cholan-24-oic acid	391.3 > 391.3	10	13.49
MDCA	Murideocycholic acid<br>: 3α,6β-Dihydroxy-5β-cholan-24-oic acid	391.3 > 391.3	20	11.50
GHDCA	Glycohyodeoxycholic acid<br>: Glyco 3α,6α-dihydroxy-5β-cholan-24-oic acid	448.3 > 74.1	45	10.10
THDCA	Taurohyodeoxycholic acid<br>: Tauro 3α,6α-Dihydroxy-5β-cholan-24-oic acid	498.3 > 79.9	65	8.42
DhLCA	Dehydrolithocholic acid<br>: 3-Oxo-5β-cholan-24-oic acid	373.3 > 373.3	10	19.73
7-KetoDCA	7-Ketodeoxycholic acid<br>: 3α,12α-Dihydroxy-7-oxo-5β-cholan-24-oic acid	405.3 > 289.2	35	10.51
7-KetoLCA	7-Ketolithocholic acid<br>: 3α-Hydroxy-7-oxo-5β-cholan-24-oic acid	435.3 > 389.2	21	13.86
12-KetoLCA	12-Ketolithocholic acid<br>: 3α-Hydroxy-12-oxo-5β-cholanoic acid	389.3 > 371.3	30	14.27
